# FTU-Seek: Foundation Model-Guided Hard-Negative Learning for Sparse Functional Tissue Unit Segmentation

**DOI:** 10.3390/biomedicines14091935

**Published:** 2026-08-28

**Authors:** Zonghao Liu, Lei Su, Jiguang Yu, Xuqing Geng, Louis Shuo Wang, Jianmin Wang, Jingfeng Liu

**Affiliations:** 1 Department of Hepatopancreatobiliary Surgery, Clinical Oncology School, Fujian Medical University, Fuzhou 350014, China; liuzonghao42@gmail.com; 2 Innovation Center for Cancer Research, Clinical Oncology School, Fujian Medical University, Fuzhou 350014, China; wangjm8605@163.com; 3 Institute of Automation, Chinese Academy of Sciences, Beijing 100190, China; sulei2023@ia.ac.cn (L.S.); gengxuqing2024@ia.ac.cn (X.G.); 4 University of Chinese Academy of Sciences, Beijing 100190, China; 5 College of Engineering, Boston University, Boston, MA 02215, USA; jyu678@bu.edu; 6 Department of Mathematics, Northeastern University, Boston, MA 02115, USA; 7 Fujian Key Laboratory of Advanced Technology for Cancer Screening and Early Diagnosis, Fujian Cancer Hospital, Fuzhou 350014, China; 8 Key Laboratory of Cancer Metabolism, National Health Commission of the People’s Republic of China, Fuzhou 350014, China

**Keywords:** functional tissue units, tertiary lymphoid structures, pathology foundation model, whole-slide imaging, deep learning, sparse object segmentation, hard negative mining, computational pathology

## Abstract

**Background/Objectives**: Functional tissue units (FTUs), including tertiary lymphoid structures (TLSs), blood vessels, and glands, encode localized immune, vascular, and epithelial organization in histopathology. Accurate quantification of these structures is important for studying tissue architecture and disease-associated tissue organization. However, FTUs are frequently sparse, heterogeneous, and surrounded by large amounts of morphologically similar background tissue, making automated segmentation in whole-slide images (WSIs) challenging. We therefore developed FTU-Seek, a pathology foundation model-guided framework that treats morphology-aware negative-patch selection as a key component of sparse FTU segmentation. **Methods**: FTU-Seek uses frozen multi-depth features from the UNI pathology foundation model to train a patch-level classifier that distinguishes FTU-containing from FTU-absent tissue. Target-absent patches are subsequently ranked according to their predicted target-containing probabilities, and the highest-scoring hard negatives are selected through a static Top*K* strategy to construct compact segmentation training sets. The framework was evaluated using five-fold cross-validation and internal test cohorts across TLS, blood-vessel, and gland segmentation tasks, with an additional independent 30-WSI held-out cohort for TLS. Positive-only, all-tissue, random-negative, and matched random Top*K* sampling strategies served as comparators. Segmentation-derived phenotypes were further explored in external TCGA cohorts. **Results**: The patch-level classifiers achieved mean validation AUCs of 95.92%, 90.11%, and 98.16% for TLS, blood vessel, and gland classification, respectively. For TLS segmentation, the pre-specified Top1000 configuration retained 27.6% of the all-tissue training workload and achieved a slide-level Dice of 76.69 ± 11.89% on the independent 30-WSI held-out cohort. Compared with matched random Top1000 sampling, it improved Dice by 3.72 percentage points (95% CI, 2.05–5.38). Blood vessel and gland segmentation achieved performance approaching all-tissue training while reducing the retained training workload by approximately one-half and one-third, respectively. Compared with matched random sampling, classifier-guided hard-negative selection produced the greatest improvements for sparse and morphologically ambiguous FTUs. Exploratory TCGA analyses further showed associations of TLS phenotypes with overall survival, vascular phenotypes with overall survival and microvascular invasion, and glandular phenotypes with clinicopathological characteristics. **Conclusions**: FTU-Seek demonstrates that pathology foundation models can support sparse FTU segmentation not only through feature representation but also through morphology-aware construction of segmentation training sets. By prioritizing informative hard negatives, the framework reduces redundant segmentation-training workload while maintaining competitive segmentation performance and supporting quantitative tissue phenotyping from routine histopathology.

## 1. Introduction

Whole-slide images (WSIs) are increasingly used not only for diagnosis, grading, and treatment planning, but also for quantitative analysis of tissue architecture and the tumor microenvironment [1,2,3]. Beyond large histological compartments, WSIs contain spatially localized microscopic structures that reflect complementary biological processes. Glandular structures encode epithelial differentiation and architectural organization [4,5,6], blood vessels reflect angiogenesis and vascular remodeling [7,8], and tertiary lymphoid structures (TLSs) represent organized antitumor immune activity [9,10,11,12]. In this study, we use the term functional tissue units (FTUs) to describe these histologically identifiable multicellular structures whose morphology, abundance, and spatial organization are linked to epithelial, vascular, or immune functions. Accurate FTU segmentation may therefore provide a practical route for extracting interpretable image-derived phenotypes from routine histopathology slides [13,14,15,16].

**Figure 1:**
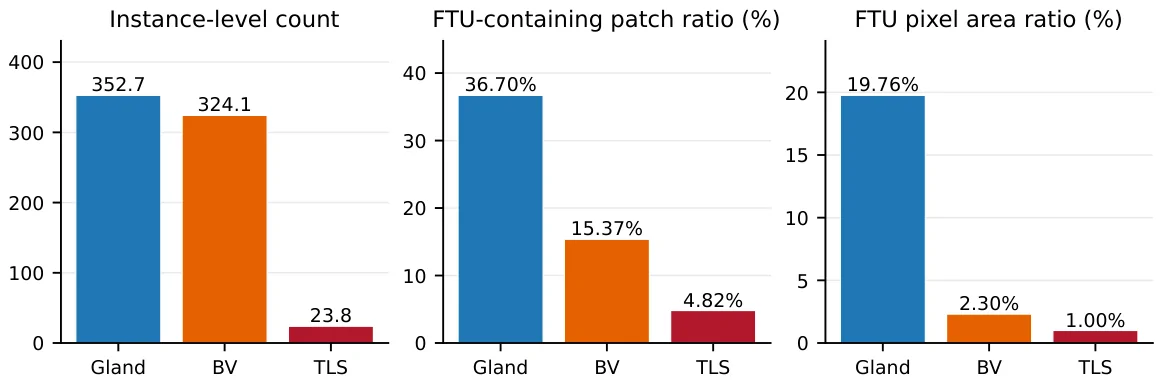
Quantitative assessment of spatial sparsity across representative functional tissue units (FTUs). Glands, blood vessels (BVs), and tertiary lymphoid structures (TLSs) were compared at the instance, patch, and pixel levels. Instance-level sparsity was assessed using FTU instance counts; the proportion of FTU-containing tissue patches measured patch-level sparsity; and pixel-level sparsity was quantified using the FTU pixel area ratio at a spatial resolution of 1 μm/pixel. This figure reports statistics calculated from the complete cohort by pooling the development and independent test sets.

**Figure 2:**
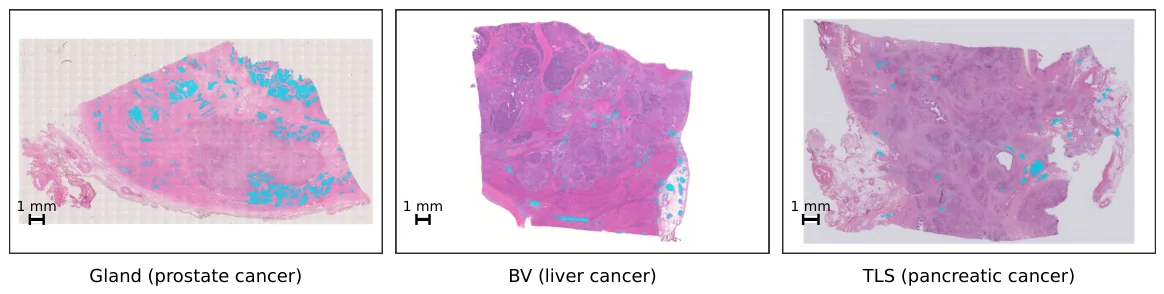
Representative spatial distributions of annotated functional tissue unit (FTU) regions in whole-slide images. Annotated FTU regions are shown in cyan for glandular structures in prostate cancer, blood vessels in liver cancer, and tertiary lymphoid structures in pancreatic cancer.

A central challenge in FTU analysis is that these structures are frequently sparse, heterogeneous, and embedded within large amounts of target-absent tissue. As shown in Figure 1, the representative FTUs studied here differ substantially at the instance, patch, and pixel levels. TLSs and blood vessels occupy only a small fraction of tissue patches and total tissue area [17,18,19,20], whereas glandular structures are more abundant but still exhibit considerable architectural and morphological variability [21,22,23]. Their spatial distributions are also highly uneven across prostate cancer, liver cancer, and pancreatic cancer WSIs, as illustrated in Figure 2. Manual delineation by pathologists is possible but labor-intensive, particularly when FTUs are small, fragmented, boundary-ambiguous, or visually confounded by surrounding tissue [24,25,26,27,28]. These characteristics make FTU segmentation a sparse-structure learning problem rather than a conventional segmentation task involving large and spatially continuous regions.

Deep learning-based WSI segmentation has achieved strong performance for relatively large and spatially continuous compartments, such as tumor parenchyma, necrosis, and stroma [29,30,31,32,33,34]. Sparse FTU segmentation imposes a fundamentally different constraint. Target-containing patches often represent only a small minority of all tissue patches, whereas the substantially larger target-absent pool contains both redundant easy negatives and a limited subset of morphologically target-like regions that can trigger false-positive (FP) predictions [35,36,37]. Training on all tissue patches is therefore inefficient because a large proportion of the negative samples contributes little additional information. Random negative sampling reduces the training workload, but may fail to retain the rare background regions that most closely resemble the target and are therefore most important for FP suppression. Accordingly, sparse FTU segmentation is not only a pixel-level prediction problem, but also a WSI-scale training-set construction problem: the model must be exposed to sufficiently informative negative tissue without being dominated by redundant background patches.

Existing imbalance-handling strategies only partially address this challenge. Loss-based methods, including class-weighted objectives, Dice-based losses, and focal loss, increase the optimization contribution of under-represented or difficult pixels [38,39,40]. Sampling-based approaches rebalance the training distribution through oversampling, undersampling, or hard-example selection. Online hard example mining prioritizes high-loss samples produced by the current model and has been influential in computer vision [41]. In computational pathology, hard-negative mining has also been applied to histopathology classification and weakly supervised WSI analysis [42,43,44]. However, many previous methods have primarily been developed for image- or slide-level classification, or for dense prediction settings in which candidate samples can be evaluated within a manageable field of view. For gigapixel WSI segmentation, repeatedly screening the complete negative pool during pixel-level training can be computationally expensive. A remaining practical challenge is to identify, before segmentation training, a compact and reproducible subset of target-absent patches enriched for morphologically confusing hard negatives. This setting is distinct from active learning, which typically selects unlabeled samples for additional expert annotation and iteratively expands the labeled training set, and from online hard-example mining, which dynamically identifies high-loss or misclassified samples during model optimization [41]. In contrast, FTU-Seek operates on an already annotated WSI patch pool. It ranks FTU-absent patches before segmentation training and constructs a compact, fixed, and reproducible training set without requesting additional annotations.

Pathology foundation models (PFMs) provide a promising basis for this selection problem. Models such as CTransPath, UNI, Virchow, and Prov-GigaPath have been used to learn transferable histomorphological representations, while vision–language models such as CONCH incorporate semantic supervision from paired biomedical text  [45,46,47,48,49,50,51]. These representations have demonstrated broad utility across cancer types and downstream prediction tasks [52]. Related studies have also explored pathology patch selection, representative region selection, and hard-example mining  [42,43,44]. Accordingly, PFMs and patch-selection strategies have been investigated in related settings.

Here, we propose FTU-Seek, a PFM-guided hard-negative learning framework for efficient segmentation of sparse FTUs in WSIs. FTU-Seek uses frozen multi-depth PFM features to train a patch-level classifier, which ranks an already annotated FTU-absent patch pool according to target-like morphology. A static per-WSI Top*K* rule is then used to select the highest-scoring negative patches and construct a compact, workload-controlled segmentation training set. Thus, FTU-Seek integrates PFM-based morphological representation, target-specific hard-negative ranking, and static training-set construction for sparse FTU segmentation.

The main contributions of this study are summarized as follows:

- We develop a PFM-guided training-set construction strategy that integrates frozen multi-depth representations, target-specific ranking of annotated FTU-absent patches, and static per-WSI Top*K* selection to reduce redundant background sampling in sparse FTU segmentation.
- We systematically evaluate classifier-guided hard-negative selection across TLS, blood-vessel, and gland segmentation tasks with distinct sparsity and morphological profiles, using positive-only, all-tissue, random-negative, and matched random Top*K* strategies to assess the trade-off between segmentation performance and retained training workload.
- We further demonstrate the downstream utility of FTU-Seek through exploratory applications to external TCGA cohorts, showing that segmentation outputs can be transformed into quantitative phenotypes describing immune, vascular, and glandular tissue organization.

Because the selected negative pool is fixed before segmentation training, this construction reduces the number of patches that must be stored, loaded, and processed during the segmentation stage. The resulting benefit is therefore an expected reduction in training-data workload and data-transfer burden, rather than a directly measured reduction in wall-clock time, GPU-memory usage, or energy consumption.

## 2. Materials and Methods

### 2.1. Study Design and Datasets

To develop FTU-Seek for precise spatial segmentation, our methodology was primarily driven by highly granular, instance-level morphological analysis. We retrospectively constructed three task-specific H&E-stained WSI cohorts from Fujian Cancer Hospital (FCH). For each task, we purposely curated a representative subset of WSIs designed to encompass a wide spectrum of tissue architectures and staining profiles, thereby maximizing morphological heterogeneity. Through exhaustive, pixel-level manual delineation of H&E-stained whole-slide images, we extracted three datasets of FTUs, establishing annotations used to dichotomize all derived image patches into FTU-containing and FTU-absent categories. Specifically, the TLS dataset comprised 2079 FTU-containing patches and 41,094 FTU-absent patches from patients with pancreatic ductal adenocarcinoma (PAAD); the gland dataset included 11,670 FTU-containing patches and 20,128 FTU-absent patches from patients with prostate adenocarcinoma (PRAD); and the blood vessel dataset included 4005 FTU-containing patches and 22,048 FTU-absent patches from patients with intrahepatic cholangiocarcinoma. Each patient contributed one WSI. For each task, WSIs were randomized into a model development set and an independent test set at a 4:1 ratio before patch extraction. The resulting cohorts contained 10 patients and 10 TLS WSIs (8 for development and 2 for internal testing), 9 patients and 9 blood-vessel WSIs (7 for development and 2 for internal testing), and 10 patients and 10 gland WSIs (8 for development and 2 for internal testing). Data partitioning was performed at the patient/WSI level and was independent of patch-level sampling. Patch extraction and subsequent positive/negative patch selection were conducted within the predefined development or test cohort, without using patch-level information to determine the split. Given the limited number of internal-test WSIs, these results were interpreted cautiously and were complemented by the independent 30-WSI held-out TLS evaluation.

The development sets—which provided a morphologically dense corpus of training patches—underwent five-fold cross-validation to optimize model hyperparameters, while the independent test sets were completely held out for final segmentation performance evaluation. To further assess the robustness of the segmentation strategies beyond the original two-slide internal test set, we constructed an additional held-out cohort of 30 TLS whole-slide images from the same institution. These WSIs were not used for model training, model selection, hyperparameter tuning, Top*K* selection, or threshold optimization. The 30-slide cohort was evaluated only after the segmentation configurations had been pre-specified based on the development-set experiments. For each method, predictions from the five cross-validation models were combined as an ensemble, and a fixed segmentation threshold of 0.5 was used for all evaluations.

To investigate the downstream utility of FTU segmentation, diagnostic WSIs and matched clinicopathological data from public TCGA cohorts were downloaded from the NIH Genomic Data Commons Data Portal (https://portal.gdc.cancer.gov) [53]. One diagnostic WSI was included per patient. Three task-specific external analyses were performed. For TLS phenotyping, TCGA-READ, TCGA-ESCA, and TCGA-STAD included 146, 148, and 326 patients, with 26, 62, and 132 deaths, respectively. TLS phenotypes were quantified in TCGA-READ, TCGA-ESCA, and TCGA-STAD to assess immune-structure abundance, size composition, and spatial organization. For vascular phenotyping, TCGA-LIHC included 337 patients with 121 deaths for overall survival (OS) analysis and the microvascular invasion (MVI) analysis. Blood-vessel phenotypes were quantified in TCGA-LIHC. For glandular phenotyping, TCGA-PRAD included 401 patients, with 395, 372, and 342 patients having available pathological T stage, surgical margin, and biochemical recurrence (BCR) information, respectively. Glandular phenotypes were quantified and evaluated in relation to Gleason grade group and the available clinicopathological endpoints described above. TCGA cases were retained when a diagnostic WSI, matched clinical endpoint, and valid tissue-level segmentation output were available. Additionally, we excluded patients with concurrent other malignancies, those who underwent resection for tumor recurrence, and those with a follow-up duration of less than one month. These analyses were designed as exploratory downstream applications of FTU-Seek-derived segmentation outputs rather than as external validation of segmentation performance or definitive clinical prediction analyses.

All procedures and protocols in this study adhered to the principles of the Declaration of Helsinki. Ethical approval for the retrospective FCH cohorts was obtained from the Ethics Committee of Fujian Cancer Hospital on 14 August 2024 (No. SQ2026-050). The requirement for informed consent was waived because the study used fully de-identified retrospective clinical data. Use of public datasets from TCGA was deemed exempt from ethics committee review because these data are de-identified and publicly available.

### 2.2. WSI Annotation and Preprocessing

All WSIs were acquired at 20× or 40× magnification. For pathological annotation, the boundaries of the target FTUs—namely TLSs, glands, and blood vessels—were manually delineated by three trained annotators using Aperio ImageScope v12.4.6 (Leica Biosystems, Wetzlar, Germany), with one annotator assigned to each FTU type. All annotations were subsequently reviewed and finalized by a senior pathologist (Y.K.) to ensure consistency with the predefined histopathological criteria described below. As annotations were not independently duplicated, inter-observer agreement was not assessed. Annotators were blinded to clinical outcome information during annotation.

- TLSs. TLSs were defined as dense aggregations of lymphocytes within non-lymphoid tissues, while loosely distributed inflammatory infiltrates lacking clear borders or cohesive architecture were excluded [54,55,56].
- Blood vessels. The annotation of vascular structures depended on the presence of an endothelial lining. Vascular contours were delineated along the outer wall, encompassing the endothelial lining and any associated structural layers (such as smooth muscle). To ensure a comprehensive vascular assessment, the annotations incorporated a diverse range of vessel types, spanning from arterioles with distinct smooth muscle layers to portal and hepatic veins, which frequently lack conspicuous smooth muscle tissue. To prevent the model from learning artifacts, tissue tearing spaces mimicking vascular lumens but lacking an endothelial lining were explicitly excluded [57,58,59].
- Glands. The boundaries of prostatic glands were precisely traced at the epithelial-stromal junction, excluding the surrounding fibromuscular stroma. These annotations encompassed both benign glands—marked by an intact bilayered architecture (inner secretory and outer basal cells) alongside intraluminal corpora amylacea—and malignant acini, characterized by basal cell loss, crowded or cribriform growth patterns, and enlarged hyperchromatic nuclei [60,61,62].

After annotation, foreground tissue regions were separated from the surrounding slide background using Otsu thresholding across all datasets [63]. To balance morphological detail with computational efficiency, tissue regions were processed at a spatial resolution of 1 μm/pixel, corresponding to approximately 10× magnification [35]. Each foreground tissue region was subsequently divided into non-overlapping image patches of 256×256 pixels. Before model input, these patches were resized to 224×224 pixels.

The same non-overlapping patch coordinates were retained throughout patch-level classification, segmentation training, validation, and testing. No additional random cropping or overlapping patch generation was performed at later stages. The image transformations applied during model input consisted only of resizing the extracted patches to the model input size, conversion to tensors, and channel-wise normalization using ImageNet mean and standard-deviation values. No random flipping, rotation, color jittering, or other data augmentation was used for either the patch classifier or the segmentation model.

Patches located entirely in slide background regions were excluded from subsequent analysis. For patch-level classification, any patch overlapping the annotated target region was labeled as target-containing, whereas patches without target overlap were labeled as target-absent. This inclusive overlap criterion was selected because the auxiliary classifier was intended to identify all patches containing potential target morphology, including partial or boundary-crossing targets, for subsequent hard-negative ranking. Using a minimum-overlap threshold could discard small or peripheral target regions and increase the risk of assigning target-containing context to the negative pool. These patch-level labels were used only for classifier training and negative-patch ranking, while segmentation training and evaluation were performed using the original pixel-level annotation masks. Thus, non-target pixels within a target-containing patch remained background during segmentation learning. The same ImageNet mean and standard-deviation normalization was used for all classifier and segmentation inputs to reduce differences in input scale across WSIs.

### 2.3. Model Development

**Figure 3:**
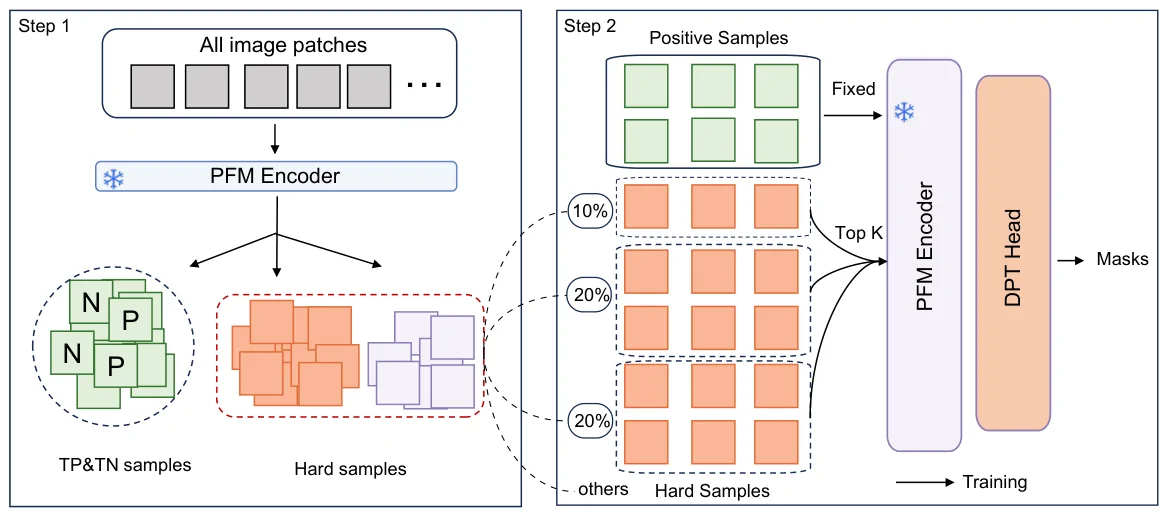
Overview of the proposed FTU-Seek framework. A patch-level classifier first ranks FTU-absent tissue patches according to their predicted FTU-containing probabilities, and classifier-derived TopK hard negatives are selected to construct compact segmentation training sets. The framework is evaluated by comparing positive-only training, all-tissue training, random TopK sampling, and classifier-guided FP-find TopK hard-negative sampling.

As shown in Figure 3, FTU-Seek was designed as a two-stage hard-negative learning framework for sparse FTU segmentation in WSIs. The framework first uses a patch-level classifier to identify FTU-absent tissue patches that are likely to be confused with FTU-containing patches.

The pathology foundation model used in FTU-Seek was UNI v1. Its encoder was kept frozen, and multi-depth UNI v1 features were used as inputs to a task-specific patch-level binary classifier. The classifier was trained to distinguish FTU-containing patches from FTU-absent patches using the development-set training WSIs within each cross-validation fold. After training, the classifier assigned each FTU-absent patch a probability of being FTU-containing. Within each WSI, FTU-absent patches were ranked according to this probability, and the highest-scoring Top*K* patches were selected as hard negatives. These selected hard negatives were combined with all FTU-containing patches to form the segmentation training set. The resulting training set was then used to optimize the segmentation decoder and prediction head, while the UNI v1 encoder remained frozen.

These classifier-derived hard negatives are then used to construct static TopK segmentation training sets. The static strategy evaluates how the number of classifier-derived hard negatives affects segmentation performance and directly compares FP-find TopK sampling with positive-only training, all-tissue training, and random TopK sampling under matched negative-sampling budgets.

#### 2.3.1. Patch-Level Training Set Definition

Stage I establishes a classifier-guided hard negative pool through patch-level FP screening. This stage consists of patch-level training set definition, classification network construction, and classifier-derived hard negative ranking. Let P denote the complete set of tissue patches extracted from the training WSIs. According to their overlap with the corresponding annotation masks, P is partitioned into a target-containing subset $P^{+}$ and a target-absent subset $P^{-}$:

$$P=P^{+}\cup P^{-},\;P^{+}\cap P^{-}=⌀.$$

A patch is assigned to $P^{+}$ if it overlaps with at least one annotated target region; otherwise, it is assigned to $P^{-}$. These patch-level labels are used to train the classifier and to identify high-risk background patches for hard negative initialization.

#### 2.3.2. Classification Network

**Figure 4:**
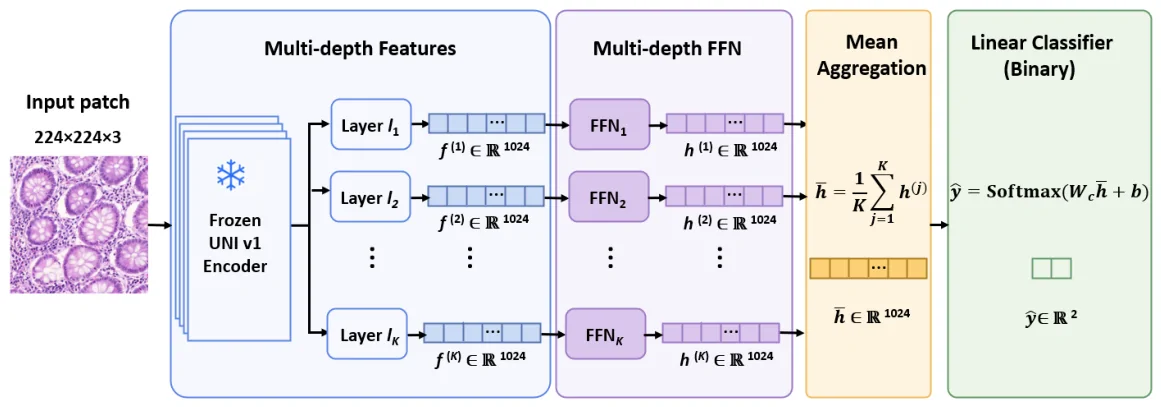
Architecture of the patch-level classification network. Multi-dimensional feature embeddings are extracted from different relative depths of the frozen UNI v1 encoder. Each feature is processed by a depth-specific feed-forward transformation head, and the transformed representations are aggregated before binary patch-level classification.

The architecture of the patch-level classification network is illustrated in Figure 4. For each input patch, feature embeddings are extracted from *K* selected relative depths of the frozen UNI v1 encoder [47]. Each depth-specific feature is independently processed by a dedicated feed-forward transformation head. The transformed features are then aggregated into a fused patch representation, which is finally mapped to a binary prediction indicating whether the patch is target-containing or target-absent.

Specifically, for an input patch $x_{i}$, the frozen UNI v1 encoder extracts *K* feature embeddings, denoted as $f_{i}^{\left(j\right)}=E_{\mathrm{UNI}}^{\left(r_{j}\right)}\left(x_{i}\right)$ for j=1,2,…,K. Here, $E_{\mathrm{UNI}}^{\left(r_{j}\right)}\left(\cdot \right)$ denotes the encoder output at the selected relative depth $r_{j}$, and $f_{i}^{\left(j\right)}\in R^{1024}$.

Each depth-specific feature is independently transformed by a feed-forward head:

$$h_{i}^{\left(j\right)}=F_{j}\;\left(f_{i}^{\left(j\right)}\right),\;j=1,2,\ldots ,K,$$

where $F_{j}\left(\cdot \right)$ denotes the feed-forward head for the *j*-th selected encoder depth. In this study, K=10, with relative encoder depths uniformly selected as 0.1, 0.2,…, 1.0. In our implementation, each head consists of two fully connected layers with a ReLU activation, expanding the feature dimension from 1024 to 4096 and projecting it back to 1024.

The transformed representations from all selected depths are fused by mean aggregation and mapped to binary class probabilities:

(1) $${\hat{y}}_{i}=\mathrm{Softmax}\left(C\left(\frac{1}{K}\sum_{j=1}^{K}h_{i}^{\left(j\right)}\right)\right),\;{\hat{y}}_{i}\in R^{2},$$

where C(·) denotes the final linear classification layer. The two output dimensions correspond to the target-absent and target-containing classes, respectively. After training, the patch-level classifier is applied to the training patch set. For each patch $x_{i}$, the predicted patch label is defined as

$${\hat{c}}_{i}=\arg\max_{c\in \{0,1\}}{\hat{y}}_{i,c},$$

where c=0 and c=1 denote the target-absent and target-containing classes, respectively.

Among the target-absent patches, those assigned high target-containing probabilities were regarded as classifier-derived hard negatives. Let $s_{i}={\hat{y}}_{i,1}$ denote the predicted target-containing probability for a negative patch $x_{i}\in P^{-}$. In the reported experiments, hard negative selection was performed independently within each WSI using the per_wsi scope, rather than on a globally pooled negative-patch set. The candidate records were ordered according to the stored within-WSI ranking, with the predicted target-containing probability used as a secondary sorting criterion. The first *K* eligible records were selected:

$$H_{K}^{-}={\mathrm{TopK}}_{x_{i}\in P^{-}}\left(s_{i}\right).$$

Here, *K* denotes the nominal number of selected classifier-derived negative patches, and K∈{100,300,500,1000} was evaluated using the same candidate values across all FTU categories. This common candidate set was chosen to enable controlled accuracy–workload comparisons under matched sampling budgets, rather than to define a prevalence-adaptive rule or a universally optimal budget. When a WSI contained fewer than *K* eligible negative patches, all available candidates were retained. Duplicate patch indices, missing coordinate entries, and patches identified as FTU-containing during coordinate checking were excluded from the final negative-patch list. If both the stored ranking and the predicted probability were tied, the original order of the merged ranking records was retained. Therefore, the final number of selected negative patches was at most *K* and could vary across WSIs. For a cross-validation fold with training-WSI set $W_{f}$, the final negative-patch count was calculated as $\sum_{w\in W_{f}}\min\left(K,N_{\mathrm{eligible},w}\right)$ after the exclusion rules described above. Each cross-validation fold used only the selected negative patches belonging to its corresponding training WSIs.

The corresponding static segmentation training set was defined as

$$T_{\mathrm{TopK}}=P^{+}\cup H_{K}^{-}.$$

This per-WSI design was used to prevent WSIs with larger tissue areas from dominating the selected negative-patch pool and to enable matched comparisons between classifier-guided and random sampling.

This setting isolates the contribution of classifier-derived hard negatives and allows direct comparison with positive-only training, all-tissue training, and random negative sampling.

#### 2.3.3. Segmentation Network

**Figure 5:**
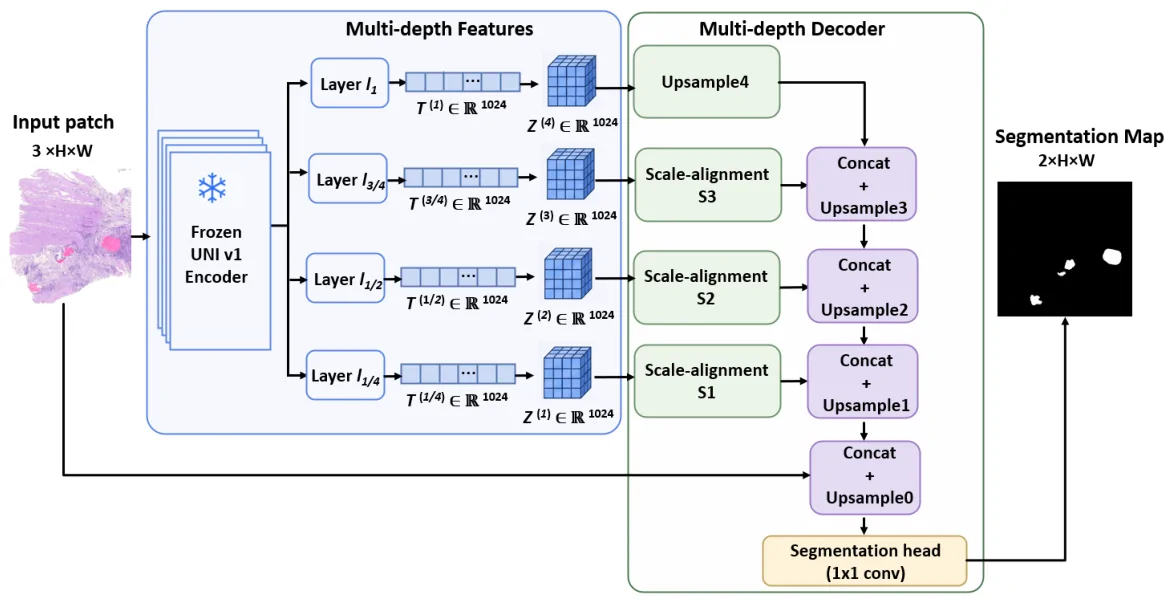
Architecture of the segmentation network. Four Transformer-layer features are extracted from the frozen UNI v1 encoder at relative depths of 1/4, 1/2, 3/4, and 1, reshaped into spatial feature maps, and progressively fused with shallow image features by a multi-scale decoder to produce dense binary segmentation predictions.

The segmentation network used in Stage II is illustrated in Figure 5. It consists of a frozen UNI v1 encoder [47] and a multi-scale feature fusion decoder inspired by dense prediction transformer designs [64]. For each input patch, multi-depth Transformer features extracted by UNI v1 are reshaped into spatial feature maps and progressively fused in the decoder. Starting from the deepest feature, the decoder gradually restores spatial resolution by integrating shallower Transformer features and a shallow convolutional representation of the original image, ultimately producing a dense two-class segmentation map.

Given an input patch $x_{i}\in R^{3\times H\times W}$, the frozen UNI v1 encoder first partitions it into non-overlapping h×w image tokens. This results in a token grid of size $H_{t}\times W_{t}$, where $H_{t}=H/h$ and $W_{t}=W/w$. To provide multi-level representations for dense prediction, we extract token features from *L* selected relative encoder depths:

$$T_{i}^{\left(j\right)}=E_{\mathrm{UNI}}^{\left(r_{j}\right)}\left(x_{i}\right),\;j=1,2,\ldots ,L,$$

where $E_{\mathrm{UNI}}^{\left(r_{j}\right)}\left(\cdot \right)$ denotes the UNI v1 output at the selected relative depth $r_{j}$. In this study, L=4 and $r_{j}\in \left\{1/4,1/2,3/4,1\right\}$. Each feature sequence contains one class token and $H_{t}\times W_{t}$ patch tokens, i.e., $T_{i}^{\left(j\right)}\in R^{(1+H_{t}W_{t})\times 1024}.$

Before decoding, the class token is removed, and the remaining patch-token subset $T_{i,\mathrm{patch}}^{\left(j\right)}$ is reshaped into a two-dimensional feature map:

$$Z_{i}^{\left(j\right)}=R\left(T_{i,\mathrm{patch}}^{\left(j\right)}\right),\;Z_{i}^{\left(j\right)}\in R^{1024\times H_{t}\times W_{t}},$$

where R(·) denotes the token-to-feature-map reshaping operation. For simplicity, the four reshaped feature maps are denoted as $Z_{1},Z_{2},Z_{3},Z_{4}$.

The decoder adopts a progressive multi-scale fusion strategy. Starting from the deepest feature $Z_{4}$, it gradually restores spatial resolution by integrating shallower encoder features. Taking the fusion with $Z_{3}$ as an example, $Z_{4}$ is first upsampled to obtain $B_{4}$, while $Z_{3}$ is transformed to the same spatial scale and channel dimension. The two features are then concatenated and further refined:

$$B_{3}=U_{3}\left(\mathrm{Concat}\left(U_{4}\left(Z_{4}\right),S_{3}\left(Z_{3}\right)\right)\right),$$

where $U_{4}\left(\cdot \right)$ denotes the bottleneck upsampling block, $S_{3}\left(\cdot \right)$ denotes the scale-alignment and channel-projection transformation for $Z_{3}$, $U_{3}\left(\cdot \right)$ denotes the fusion-and-upsampling block, and Concat(·,·) denotes channel-wise concatenation.

The same fusion procedure is applied to the remaining encoder features. Specifically, $B_{3}$ is fused with the transformed $Z_{2}$ to produce $B_{2}$, and $B_{2}$ is further fused with the transformed $Z_{1}$ to obtain $B_{1}$. In our implementation, the decoded feature resolution is progressively restored from the token-grid resolution $H_{t}\times W_{t}$ to the original input resolution H×W.

In parallel, the original input image is processed by a shallow convolutional branch $S_{0}\left(\cdot \right)$ to preserve local texture and boundary information. The final segmentation prediction is obtained by concatenating the decoded feature $B_{1}$ with the shallow image feature $S_{0}\left(x_{i}\right)$ and passing the fused representation through the segmentation head:

$${\hat{Y}}_{i}=\mathrm{Softmax}\left(H\left(\mathrm{Concat}\left(B_{1},S_{0}\left(x_{i}\right)\right)\right)\right),\;{\hat{Y}}_{i}\in R^{2\times H\times W},$$

where H(·) denotes the final convolutional prediction head, and Concat(·,·) denotes channel-wise concatenation. The two output channels correspond to the non-target and target classes, respectively.

During training, the UNI v1 encoder remains frozen, while the downstream decoding modules and segmentation head are optimized. This design leverages pretrained pathology representations while allowing the task-specific prediction layers to adapt to the segmentation targets. In the present experiments, the static TopK setting was used as the primary FTU-Seek configuration. This choice keeps the training protocol reproducible and isolates the effect of classifier-guided hard-negative selection from additional segmentation-stage re-mining. The candidate values K∈{100,300,500,1000} were predefined exploratory operating points selected to cover progressively broader hard-negative budgets, from highly compact training sets to more extensive negative coverage. They were not adopted from a specific prior study and were not intended to define a universally optimal or prevalence-adaptive budget.

#### 2.3.4. Optimization Objectives and Training Protocol

The patch-level classifier was optimized using cross-entropy loss. Let $y_{i}=\left[y_{i,0},y_{i,1}\right]$ denote the one-hot patch-level ground-truth label, and let ${\hat{y}}_{i}=\left[{\hat{y}}_{i,0},{\hat{y}}_{i,1}\right]$ denote the predicted class probabilities obtained from Equation (1). The classification loss is defined as

$$L_{\mathrm{cls}}=-\frac{1}{N}\sum_{i=1}^{N}\sum_{c=0}^{1}y_{i,c}\log{\hat{y}}_{i,c},$$

where *N* is the number of training patches in a mini-batch, and c=0 and c=1 correspond to the target-absent and target-containing classes, respectively.

To address the foreground–background imbalance inherent to sparse target segmentation, Dice–Focal loss was adopted for model optimization [39,65,66]: $L_{\mathrm{seg}}=L_{\mathrm{Dice}}+L_{\mathrm{Focal}}.$

Let $p_{n}\in \left[0,1\right]$ denote the predicted target-class probability of the *n*-th pixel and $g_{n}\in \left\{0,1\right\}$ denote the corresponding ground-truth label. The Dice loss is defined as

$$L_{\mathrm{Dice}}=1-\frac{2\sum_{n=1}^{M}p_{n}g_{n}+\epsilon }{\sum_{n=1}^{M}p_{n}+\sum_{n=1}^{M}g_{n}+\epsilon },$$

where *M* is the total number of pixels involved in the loss computation and ϵ is a small constant for numerical stability.

The focal loss is defined as

$$L_{\mathrm{Focal}}=-\frac{1}{M}\sum_{n=1}^{M}\left[\alpha g_{n}{\left(1-p_{n}\right)}^{\gamma }\log p_{n}+\left(1-\alpha \right)\left(1-g_{n}\right)p_{n}^{\gamma }\log\left(1-p_{n}\right)\right],$$

where α and γ denote the balancing and focusing parameters, respectively. Five-fold cross-validation was performed on the development set at the WSI level. In each fold, models were trained using the corresponding training split, and model selection was performed on the validation split. For classifier-guided hard negative strategies, the patch-level classifier was trained within the same fold to rank target-absent patches, and the resulting TopK hard negatives were used to construct the segmentation training set.

The segmentation model was trained using a Dice–Focal loss and a ReduceLROnPlateau learning rate scheduler. For each fold, the model with the best validation performance was retained and evaluated on the independent test set.

#### 2.3.5. Comparative Patch Construction Strategies

To assess the contribution of classifier-guided hard negative selection, we compared the following patch construction strategies:

- Positive-only training. This strategy trains the segmentation model only with target-containing patches: $T_{\mathrm{pos}}=P^{+}$.
- All-tissue training. This strategy uses all available tissue patches: $T_{\mathrm{all}}=P^{+}\cup P^{-}$.
- Random negative sampling.
  This strategy retains all target-containing patches and randomly selects a target-absent subset $R^{-}$ from $P^{-}$. For the matched random-*K* experiments, eligible negative patches were sampled independently within each WSI without replacement using $\min(K,N_{\mathrm{eligible}})$ candidates, where $N_{\mathrm{eligible}}$ denotes the number of eligible negative patches available for that WSI. Thus, matched random-*K* sampling used the same per-WSI negative-patch budget as the corresponding FTU-Seek Top*K* configuration, but without classifier-based ranking. The corresponding segmentation training set was
  $$T_{\mathrm{rand}}=P^{+}\cup R^{-}.$$
- Static FP-find TopK sampling. This strategy trains the model with all positive patches and the classifier-derived TopK hard negatives: $T_{\mathrm{TopK}}=P^{+}\cup H_{K}^{-}$. This setting evaluates whether high-risk negative patches selected by the patch-level classifier are more informative than randomly selected negatives.

### 2.4. Outcomes

The performance of the deep learning framework was evaluated across two distinct dimensions: spatial segmentation accuracy and patch-level classification performance. To assess the spatial overlap and delineation accuracy of the segmentation network, the Dice similarity coefficient (DSC) was utilized as the primary metric [38]. DSC quantifies the pixel-level agreement between the model’s predicted output masks and the manual ground truth annotations, providing a rigorous measure of morphological fidelity:

$$\mathrm{DSC}=\frac{2\mathrm{TP}}{2\mathrm{TP}+\mathrm{FP}+\mathrm{FN}}.$$

For the patch-level classification performance, a confusion matrix was constructed to tally true positives (TPs), true negatives (TNs), false positives (FPs), and false negatives (FNs). The principal metric for this phase was the area under the receiver operating characteristic curve (AUC), which provides a threshold-independent measure of discriminative ability. Additionally, several secondary metrics were derived from the confusion matrix to provide a comprehensive assessment. Sensitivity (recall) and specificity were calculated to evaluate the capacity to correctly identify positive and negative cases:

$$\mathrm{Sensitivity}=\frac{\mathrm{TP}}{\mathrm{TP}+\mathrm{FN}},\;\mathrm{Specificity}=\frac{\mathrm{TN}}{\mathrm{TN}+\mathrm{FP}}.$$

Positive predictive value (PPV) and negative predictive value (NPV) assessed the reliability of the classifier predictions:

$$\mathrm{PPV}=\frac{\mathrm{TP}}{\mathrm{TP}+\mathrm{FP}},\;\mathrm{NPV}=\frac{\mathrm{TN}}{\mathrm{TN}+\mathrm{FN}}.$$

Furthermore, overall accuracy and the F1-score—the harmonic mean of precision and sensitivity—were computed to measure holistic performance [67], particularly addressing potential class imbalance:

$$\mathrm{Accuracy}=\frac{\mathrm{TP}+\mathrm{TN}}{\mathrm{TP}+\mathrm{TN}+\mathrm{FP}+\mathrm{FN}},\;F1-\mathrm{score}=2\times \frac{\mathrm{PPV}\times \mathrm{Sensitivity}}{\mathrm{PPV}+\mathrm{Sensitivity}}.$$

For downstream analysis, segmentation outputs were converted into slide- or patient-level FTU phenotypes. TLS features included TLS area density (ratio of TLS area to total tissue area), TLS count density (number of TLS per unit area), size-stratified TLS densities (small/medium/large by area tertiles), hotspot TLS density, and nearest-neighbor clustering indices (observed mean nearest-neighbor distance divided by random expectation). Blood-vessel features included total and filtered BV density, vessel count density, size-stratified vessel densities, and hotspot vascular density measures. Gland features included gland density, gland count density, size-stratified gland densities, and shape descriptors such as area, perimeter, circularity, solidity, and eccentricity. OS was used for TLS and BV survival analyses when survival information was available. In LIHC, the distributions of vascular characteristics were compared between MVI-positive and MVI-negative patients. For PRAD, glandular features were evaluated primarily against pathology-related endpoints and BCR status; BCR was analyzed as a binary endpoint because reliable BCR-specific censoring times were not available for non-recurrent cases. Additionally, we evaluated the differences in gland features across patients with high versus low Gleason scores, different pT stages, and resection margin statuses (R0 vs. R1).

### 2.5. Implementation Details

All model training experiments were implemented in PyTorch and performed on an NVIDIA RTX PRO 6000 (NVIDIA Corporation (Santa Clara, CA, USA)) Blackwell Workstation Edition GPU with 97,887 MiB of memory. Although two GPUs were available on the workstation, each training run was executed on a single GPU using the specified CUDA device. To improve reproducibility, Python, NumPy, PyTorch, and CUDA random seeds were fixed, and deterministic cuDNN settings were enabled.

The patch-level classifier was trained in the FP-find classification pipeline. For each FTU task and cross-validation fold, frozen UNI v1 embeddings were extracted from ten relative encoder depths (0.1,0.2,…,1.0). Each 1024-dimensional depth-specific feature was passed through a two-layer feed-forward transformation head with ReLU activation, expanded to 4096 dimensions and projected back to 1024 dimensions. The transformed multi-depth features were averaged and passed to a final linear classification layer to predict target-absent versus target-containing patch labels. The classifier was optimized using cross-entropy loss and AdamW with an initial learning rate of $5\times 10^{-5}$, weight decay of $5\times 10^{-6}$, batch size of 128, and a maximum of 500 epochs. A ReduceLROnPlateau scheduler was applied to the validation AUC, and early stopping was used with a patience of 15 epochs. For each fold, the checkpoint with the highest validation AUC was selected as the validation-best classifier. This classifier was then applied to FTU-absent training patches to compute the predicted FTU-containing probability. These probabilities were used to rank candidate hard negatives, and the top-ranked FTU-absent patches were exported for downstream static TopK segmentation training.

The segmentation model was trained within the FTU-Seek segmentation pipeline. For each FTU task, five-fold cross-validation was performed at the WSI level on the development set, and the independent test set was held out for final evaluation. The segmentation network used a frozen UNI v1 encoder and a trainable residual feature-fusion decoder. Four encoder depths (1/4, 1/2, 3/4, and 1) were used for dense prediction, and the corresponding token features were reshaped into spatial feature maps before decoder fusion. Input image patches were resized to 224×224 pixels and normalized before model input. For classifier-guided FTU-Seek sampling, the segmentation training set contained all FTU-containing patches and the classifier-ranked FTU-absent hard negatives selected by static TopK sampling within each WSI. Matched random TopK experiments used the same number of negative patches per WSI but selected them randomly from the FTU-absent pool.

During segmentation training, only the decoder and prediction head were updated, whereas all UNI v1 encoder parameters remained frozen. The trainable parameters were optimized using AdamW with an initial learning rate of $5\times 10^{-5}$, weight decay of $5\times 10^{-6}$, batch size of 32, and a maximum of 50 epochs. Mixed-precision training was enabled when CUDA was available using automatic casting and gradient scaling. The segmentation objective combined multiclass Dice loss and focal loss. In the implementation, the focal term used class weights of 0.25 and 0.75 with a focusing parameter of γ=2.0, and the final loss was defined as $L_{\mathrm{seg}}=L_{\mathrm{Dice}}+20L_{\mathrm{Focal}}$. A ReduceLROnPlateau scheduler monitored the validation mean Dice coefficient and reduced the learning rate with a factor of 0.95, patience of 5 epochs, cooldown of 2 epochs, and minimum learning rate of $1\times 10^{-7}$. Validation was performed after each training epoch, and early stopping was applied with a patience of 5 epochs. For each fold, the checkpoint with the highest validation mean Dice was retained and subsequently evaluated on the independent test set.

For reproducibility, the primary experiments were conducted using random seed 1, and repeated random-baseline experiments used seeds 1–5. The WSI-level fold assignments and train–validation–test split metadata were fixed before model training and were stored in the corresponding split files. Experiments were run in Python 3.11.15 using PyTorch 2.8.0+cu128, torchvision 0.23.0+cu128, CUDA 12.8, NumPy 1.26.4, pandas 2.3.3, SciPy 1.12.0, h5py 3.15.0, timm 1.0.20, einops 0.8.1, Pillow 12.2.0, Matplotlib 3.10.7, and seaborn 0.13.2. The pathology foundation model was the publicly released UNI v1 model from the Mahmood Lab. The source code, split metadata, configuration files, and model weights that can be legally shared will be made publicly available upon publication.

During inference, the foreground probability was obtained from the foreground class of the two-class softmax output. A fixed threshold of 0.5 was used to convert the probability map into a binary foreground mask, with pixels having foreground probability ≥0.5 classified as positive. Predictions outside the foreground tissue mask were set to zero. Patch-level predictions were mapped back to their original WSI coordinates for slide-level mask visualization and downstream tissue phenotyping. For Dice calculation, pixel-level true-positive, false-positive, and false-negative counts were accumulated across all evaluated patches within each WSI. Thus, the reported Dice was not obtained by averaging patch-level Dice values. No connected-component filtering, hole filling, minimum-instance-size filtering, or other morphological post-processing was applied before Dice calculation.

### 2.6. Statistical Analysis

Segmentation and classification metrics on the development set and internal test set were summarized as mean ± standard deviation across five cross-validation folds. For the multi-seed experiments, the original run used seed 1, and four additional random-baseline runs using seeds 2–5 were subsequently performed. Random-baseline results were summarized across all five seeds (1–5) using the mean and standard deviation. These standard deviations quantify variability caused by random sampling and stochastic training across seeds. Because the original FP-find experiments were conducted using seed 1, the multi-seed analysis was interpreted primarily as an assessment of the stability of the random-sampling baselines rather than as a direct estimate of FP-find seed robustness.

For the 30-slide held-out TLS evaluation, Dice scores were calculated independently for each WSI and summarized as mean ± standard deviation across slides. For each paired comparison, the paired difference was calculated as the Dice score of FTU-Seek minus that of the comparator strategy. Two-sided 95% confidence intervals for the mean paired differences were calculated using the Student’s *t* distribution with 29 degrees of freedom. Paired *t*-tests and Wilcoxon signed-rank tests were performed using the WSI as the pairing unit. The comparisons included matched random Top*K* strategies at K=100, 300, 500, and 1000, as well as the representative Top1000 FTU-Seek strategy versus all-tissue and random-balanced training. All formal paired analyses were restricted to the independent 30-WSI held-out TLS cohort. The internal test cohort contained only two WSIs and was therefore retained for descriptive comparison rather than formal hypothesis testing. Predictions for the held-out cohort were generated using five-fold probability ensembles, in which foreground probabilities from the five-fold models were averaged before thresholding. The reported *p*-values were nominal two-sided values.

For segmentation, the Dice coefficient was reported on both validation folds and independent test sets, and training workload was calculated as the proportion of retained training patches relative to all-tissue training within each cohort.

For each evaluation WSI, pixel-level predictions and reference masks were processed patch by patch, while the numbers of true-positive, false-positive, and false-negative pixels were accumulated across all evaluated patches from that WSI. The WSI-level Dice coefficient was then calculated as

$${\mathrm{Dice}}_{\mathrm{WSI}}=\frac{2TP_{\mathrm{WSI}}}{2TP_{\mathrm{WSI}}+FP_{\mathrm{WSI}}+FN_{\mathrm{WSI}}}.$$

Thus, the reported Dice was not obtained by averaging patch-level Dice coefficients and was not an instance-level metric. Because the extracted patches were non-overlapping, this accumulation corresponds to pixel-level evaluation over the complete WSI tissue region without double-counting overlapping patches.

For the original development and internal-test experiments, Dice values were summarized across the five cross-validation folds. For the held-out TLS analysis, one WSI-level Dice value was obtained for each of the 30 WSIs, and these WSI-level values were used for the paired comparisons and confidence interval calculations.

For downstream survival analyses, OS was defined as the interval from diagnosis or surgery to death from any cause, with surviving patients censored at last follow-up. A total of 60 TLS features were evaluated separately in each of TCGA-READ, TCGA-ESCA, and TCGA-STAD, while 36 vascular features were evaluated in TCGA-LIHC. For TLS analyses, pairwise Wilcoxon rank-sum tests were used for descriptive cross-cohort comparison of TLS morphology and spatial organization, and associations with OS were evaluated using Cox proportional hazards models with individual TLS features entered as continuous variables. Features were standardized to facilitate comparison across different measurement scales, and hazard ratios (HRs) with 95% confidence intervals (CIs) were reported per 1-standard-deviation (SD) increase. To account for multiple feature-wise survival analyses, the Benjamini–Hochberg false discovery rate (FDR) procedure was applied separately within each TLS cohort across the 60 Cox regression tests performed in that cohort. Kaplan–Meier analyses were used as exploratory visualizations of selected TLS phenotypes. For exploratory cut-point analysis, candidate thresholds were searched under minimum group-size constraints to avoid extreme boundary splits, and the threshold with the smallest log-rank *p* value was retained for visualization.

For LIHC, vascular phenotypes were further examined in relation to OS and MVI. For survival analysis, selected features were dichotomized using the exploratory data-derived cut points described above and evaluated using Cox proportional hazards models, with HRs and 95% CIs reported for the high- versus low-feature groups. Because these cut points were outcome-derived, the corresponding survival analyses were considered exploratory and were not interpreted as defining validated prognostic thresholds. Multivariable Cox regression was used to assess whether the vascular phenotype remained associated with OS after adjustment for available clinical covariates. The proportional-hazards assumption was assessed using a feature-by-log-transformed-time interaction term, and multicollinearity among predictors was evaluated using variance inflation factors (VIFs). For MVI, all 36 vascular features were compared between patients with and without MVI using the Wilcoxon rank-sum test and summarized as median and interquartile range (IQR), with rank-biserial correlation reported as the effect size. The Benjamini–Hochberg FDR procedure was applied across the 36 feature-wise MVI comparisons within TCGA-LIHC. Features of interest were subsequently evaluated in multivariable logistic regression as continuous variables, with associations reported as adjusted odds ratios (ORs) and corresponding 95% CIs. Available clinical covariates were included without univariable statistical pre-screening, except for tumor stage, which was excluded because of its close relationship with vascular invasion and the potential for information overlap. No missing data were present among the 337 patients included in the analysis. Continuous vascular predictors were Z-standardized before regression, and associations were reported as adjusted ORs with 95% CIs per 1-SD increase. Multicollinearity was assessed using VIFs, and potential nonlinearity of the vascular phenotype–MVI association was examined using restricted cubic splines (RCSs). The multivariable regression analyses were used to assess adjusted associations rather than to develop clinical prediction models.

Similarly, for PRAD glandular analyses, 40 glandular features were evaluated according to Gleason score (≤7 vs. >7), pathological T stage (T2 vs. T3/T4), surgical margin status (R0 vs. R1), and BCR status (No vs. Yes). Continuous glandular features were compared between groups using the Wilcoxon test and summarized as median and interquartile range (IQR). Effect sizes were reported using rank-biserial correlation. The Benjamini–Hochberg FDR procedure was applied separately within each clinicopathological endpoint, corresponding to 40 feature-wise comparisons for Gleason score, 40 for pathological T stage, 40 for surgical margin status, and 40 for BCR status. All statistical tests were two-sided, and p<0.05 was considered nominally significant. For the exploratory TLS survival analyses and the vascular and glandular feature-comparison analyses, FDR-adjusted p<0.10 was used as an exploratory multiple-testing threshold. The downstream analyses were designed as proof-of-concept evaluations of associations between segmentation-derived phenotypes and clinicopathological characteristics rather than as confirmatory biomarker-validation analyses, and the resulting clinical associations were therefore considered hypothesis-generating.

## 3. Results

### 3.1. Dataset Characteristics

As shown in Table 1, the three FTU segmentation cohorts differed substantially in target abundance and spatial sparsity at the instance, patch, and pixel levels. At the instance level, the development sets contained 133 TLSs, 2508 blood vessels, and 3074 glands, corresponding to mean numbers of 16.62, 358.29, and 384.25 instances per WSI, respectively. A similar pattern was observed in the test sets, indicating that TLSs were considerably less frequent than blood vessels and glands.

The patch-level statistics further demonstrated pronounced class imbalance. In the TLS development set, only 1322 of 33,752 tissue patches contained annotated TLS regions, yielding an FTU-containing patch ratio of 3.92%. The corresponding ratio increased to 8.04% in the test set, with 757 positive patches among 9421 tissue patches. For BV segmentation, 3422 of 20,831 development patches and 583 of 5222 test patches contained target regions, corresponding to ratios of 16.43% and 11.16%, respectively. In contrast, glands were more densely distributed, with FTU-containing patch ratios of 36.86% in the development set and 35.92% in the test set.

This difference was also evident at the pixel level. TLS regions accounted for only 0.65% of the tissue area in the development set and 2.32% in the test set. BV pixels represented 2.46% and 1.67% of the tissue area, respectively, whereas gland regions occupied substantially larger proportions of 18.91% and 23.86%. Collectively, these statistics show that TLS segmentation represents the most spatially sparse and imbalanced task, BV segmentation exhibits moderate sparsity, and gland segmentation contains comparatively abundant target regions at both patch and pixel levels.

As summarized in Table 2 and Table 3, external TCGA cohorts were used for exploratory downstream application across the three FTU segmentation tasks. For TLS, OS analyses were conducted in the TCGA-READ, TCGA-ESCA, and TCGA-STAD cohorts. For BV, vascular phenotypes in TCGA-LIHC were evaluated in relation to OS and MVI. For gland segmentation, the TCGA-PRAD cohort was evaluated against multiple clinicopathological endpoints, including Gleason score, pathological T stage, surgical margin status, and BCR status. These cohorts enabled an exploratory assessment of whether the automatically quantified FTU features were associated with patient prognosis and clinically relevant pathological characteristics across different cancer types.

### 3.2. Patch-Level Classifier Performance

Before segmentation training, we evaluated whether the patch-level classifier could distinguish FTU-containing patches from FTU-absent tissue patches and provide a reliable ranking score for hard-negative selection.

**Figure 6:**
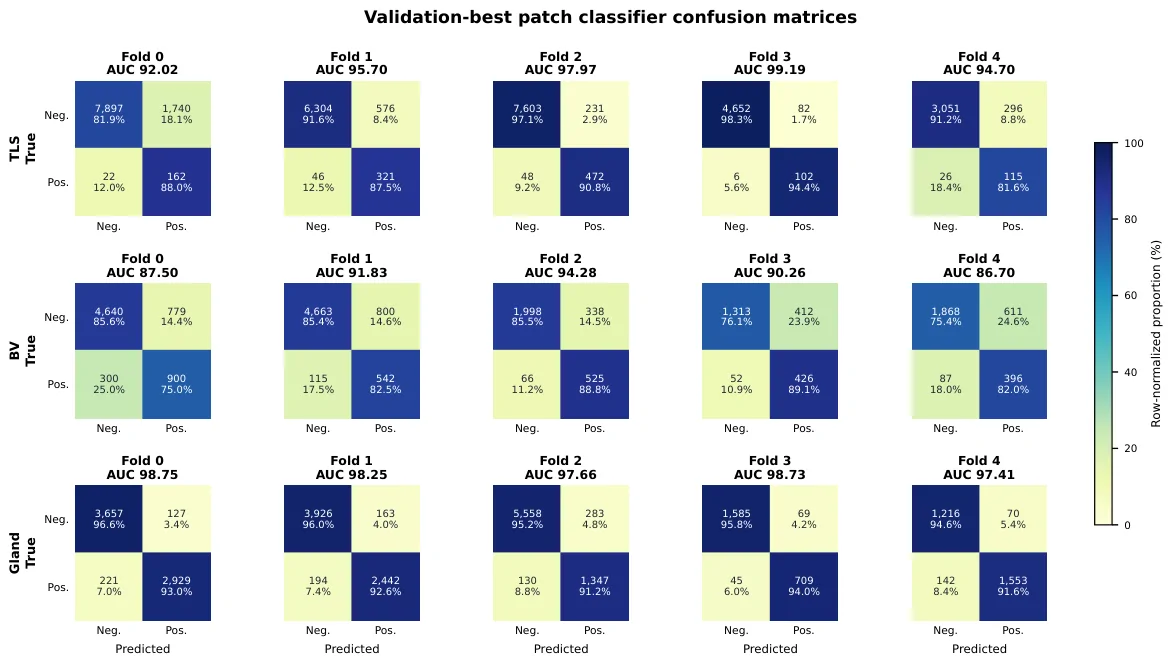
Confusion matrices detailing the patch-level classification performance of FTUs across 5-fold cross-validation. The confusion matrices illustrate the validation-best prediction results for three independent FTU segmentation cohorts: tertiary lymphoid structure (TLS, top row), blood vessel (BV, middle row), and gland (bottom row). Columns correspond to the validation set results from Fold 0 through Fold 4, demonstrating the robust generalization of the deep learning framework. Within each matrix, the absolute counts of image patches are presented alongside the row-normalized proportions, detailing the true positive rate (sensitivity) and true negative rate (specificity). The color intensity of the heatmap corresponds to these row-normalized percentages. The overall area under the receiver operating characteristic curve (AUC) achieved for each specific fold and classification task is annotated above the respective subplot.

As summarized in Table 4 and visualized in Figure 6, the validation-best patch classifiers achieved consistently strong discrimination across the three FTU tasks, although their error profiles differed according to target prevalence and morphological complexity. The TLS classifier achieved a mean AUC of 95.92±2.81%, with a sensitivity of 88.46±4.74% and a specificity of 92.01±6.46%. The corresponding confusion matrices showed true-positive rates ranging from 81.6% to 94.4% and true-negative rates ranging from 81.9% to 98.3%. Its high NPV of 99.48±0.30% indicates reliable exclusion of TLS-absent patches, whereas the lower and more variable PPV of 38.97±23.04% reflects the pronounced rarity of TLS-containing patches and the resulting sensitivity of precision to class prevalence.

The BV classifier achieved a mean AUC of 90.11±3.11%, with a sensitivity of 83.49±5.82% and a specificity of 81.60±5.36%. Across the five folds, true-positive rates ranged from 75.0% to 89.1%, while true-negative rates ranged from 75.4% to 85.6%. The relatively high mean FPR of 18.40±5.36%, particularly in Folds 3 and 4, indicates that nonvascular tissue structures with vessel-like morphology were more frequently classified as positive. Nevertheless, the classifier maintained a high NPV of 96.01±1.39%, supporting its utility for excluding patches unlikely to contain vascular structures.

Among the three tasks, the gland classifier demonstrated the strongest and most stable performance, achieving a mean AUC of 98.16±0.61%, sensitivity of 92.49±1.12%, and specificity of 95.64±0.80%. The confusion matrices showed consistently high true-positive rates of 91.2–94.0% and true-negative rates of 94.6–96.6%, together with a low mean FPR of 4.36±0.80%. Its PPV and NPV reached 91.81±5.47% and 94.82±3.26%, respectively, indicating reliable identification of both gland-containing and gland-absent patches. Collectively, these results demonstrate that the patch classifiers provided effective initial screening for all three FTU segmentation tasks, with gland classification showing the greatest stability, TLS classification being primarily affected by extreme target sparsity, and BV classification exhibiting greater susceptibility to morphologically similar background regions.

**Figure 7:**
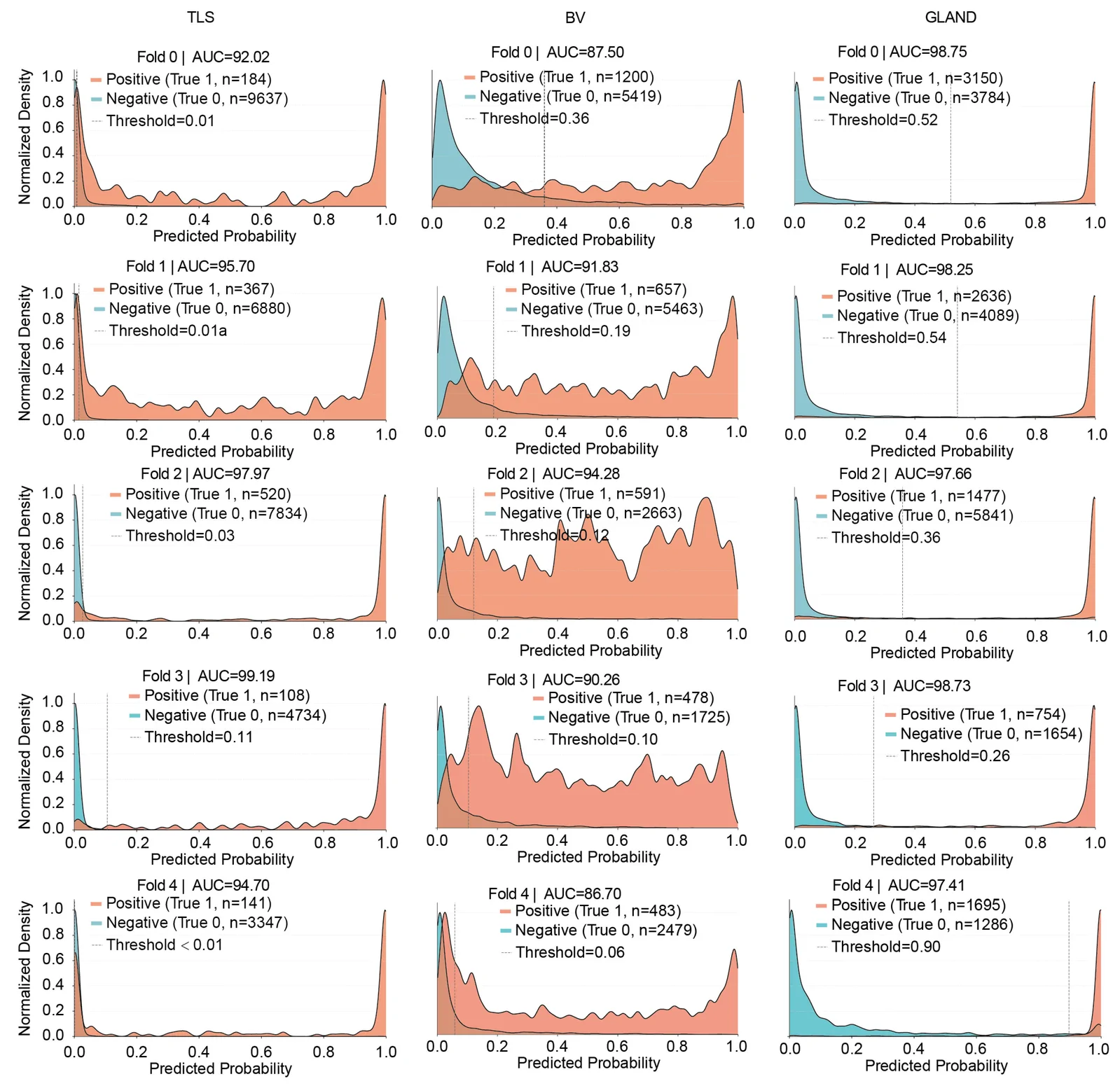
Validation-best predicted probability distributions of the patch-level classifiers across five-fold cross-validation. Columns correspond to the TLS, BV, and gland cohorts, whereas rows represent Fold 0 through Fold 4. Each panel shows the normalized density distributions of the predicted FTU-containing probabilities for annotated FTU-containing patches (orange; True 1) and FTU-absent patches (teal; True 0); *n* indicates the number of patches in each class. The black dashed vertical line indicates the fold-specific decision threshold, whose numerical value is provided within each panel. The validation AUC obtained from the best-performing checkpoint is reported in the corresponding panel title.

As shown in Figure 7, the predicted probability distributions revealed distinct task-specific patterns of class separability across the five validation folds. For TLS, FTU-absent patches were predominantly concentrated near a predicted probability of 0, whereas TLS-containing patches generally accumulated near 1.0. This pronounced bimodal separation was particularly evident in Folds 2 and 3, which achieved AUCs of 97.97% and 99.19%, respectively. Nevertheless, a subset of TLS-containing patches received low predicted probabilities in several folds, resulting in relatively low fold-specific decision thresholds ranging from below 0.01 to 0.11. This pattern is consistent with the extreme rarity and heterogeneous appearance of TLS-containing patches.

The BV classifiers exhibited substantially greater overlap between the positive and negative probability distributions. Although most BV-absent patches remained concentrated toward low probabilities, BV-containing patches were broadly distributed across the full probability range, particularly in Folds 2–4. The fold-specific thresholds consequently varied from 0.06 to 0.36. This less distinct separation agrees with the lower AUC and higher false-positive rates observed for BV classification, suggesting that vessel-containing patches were more difficult to distinguish from morphologically similar background tissue.

In contrast, the gland classifiers demonstrated the clearest and most consistent separation between classes. Across all folds, gland-absent patches were sharply concentrated near 0, while gland-containing patches formed prominent peaks near 1.0, with only limited overlap between the two distributions. This pattern was reflected in the consistently high fold-wise AUCs of 97.41–98.75%. Although the selected decision thresholds varied from 0.26 to 0.90, the strong separation of the two distributions resulted in stable sensitivity and specificity across folds. Overall, the probability distributions corroborate the quantitative results, demonstrating strong class separability for gland and TLS classification, while highlighting the greater ambiguity of the BV task.

**Figure 8:**
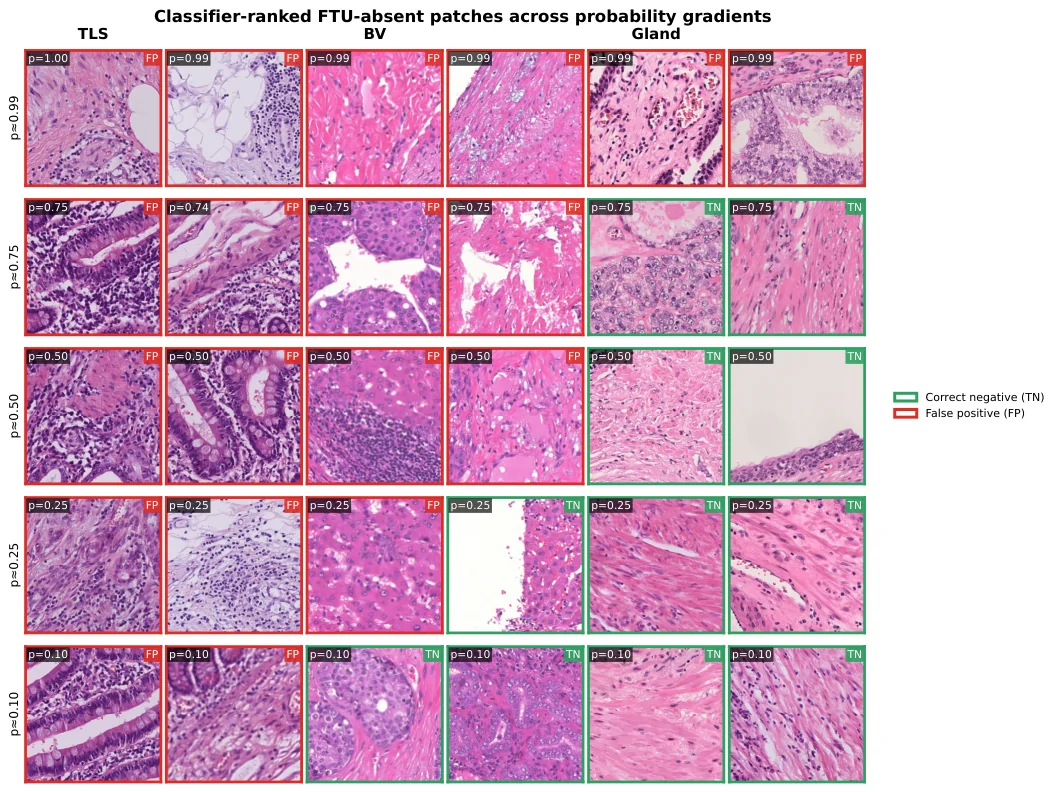
Representative classifier-ranked FTU-absent patches across predicted probability gradients. For each cohort, two FTU-absent tissue patches are shown at each approximate predicted FTU-containing probability level. Patch borders indicate classification correctness according to the validation-best cutoff of the corresponding fold: green denotes correctly classified negative patches (TN), whereas red denotes false-positive patches (FP) misclassified as FTU-containing. The examples illustrate how the patch-level classifier assigns high probabilities to morphologically confusing background regions, which are subsequently used as hard negatives for segmentation training.

Representative hard-negative examples are shown in Figure 8. TLS hard negatives often contained dense inflammatory or stromal regions adjacent to adipose or gland-like spaces, BV hard negatives included lumen-like or collagen-rich structures, and gland hard negatives contained epithelial or stromal patterns resembling gland boundaries. These examples support the use of classifier-derived probability scores to prioritize FTU-absent patches that are visually similar to target-containing regions.

### 3.3. Segmentation Performance and Training-Data Workload

Table 5, Table 6 and Table 7 summarize the segmentation accuracy and training workload of different patch construction strategies. Overall, positive-only training produced the weakest or near-weakest performance, indicating that FTU-absent tissue is necessary for suppressing false-positive predictions. All-tissue training provided the strongest reference performance in most settings but required the complete tissue-patch set. In contrast, classifier-guided FP-find Top*K* sampling retained only a fraction of the training data while preserving much of the independent-test performance.

**Table 5:** TLS Dice performance (%) across hard-negative budgets and evaluation cohorts.

	Development-Set Validation		Internal Test		Held-Out TLS Cohort	
K	FTU-Seek	Random (5 Seeds)	FTU-Seek	Random (5 Seeds)	FTU-Seek	Random (5 Seeds)
100	68.75	66.62±3.03	85.08	79.86±3.45	73.99	68.87±1.95
300	73.64	68.82±1.63	84.69	80.88±1.55	76.54	69.92±2.74
500	72.63	70.28±0.79	85.14	81.66±1.24	75.19	70.33±2.00
1000	76.91	73.54±2.25	85.12	83.92±0.54	76.69	73.48±0.62

For each random seed, the model was trained using five-fold cross-validation. The fold-level performance estimates were first averaged within each seed to obtain a seed-level mean, and the final Random value was then calculated as the mean across five random seeds. Thus, the Random entries summarize performance hierarchically across five folds and five seeds. For the compact budget comparison, FTU-Seek entries correspond to the original seed-1 experiments and are reported as fold-mean performance; the fold-level variability of the representative Top1000 configuration is reported in Table 8. For the held-out TLS cohort, each fold-specific model was evaluated on the same 30 held-out WSIs, and the resulting performance estimates were summarized within folds, then across folds within each seed, and finally across the five random seeds. These values therefore represent aggregate performance summaries and are distinct from the WSI-level paired analyses reported separately in Table 9.

**Table 6:** Comparison of representative training-set construction strategies for blood-vessel and gland segmentation. Values are Dice coefficients (%, mean ± SD) across five folds.

Task	Strategy	Workload (%)	Validation Dice	Internal-Test Dice
BV	Positive-only	16.4	47.80±4.80	32.14±3.98
	Random-balanced (1:1)	32.8	57.84±4.06	48.53±8.16
	All-tissue	100.0	62.46±6.63	60.38±9.76
	FTU-Seek	50.0	60.11±7.43	57.68±6.88
Gland	Positive-only	36.9	80.78±11.30	80.01±2.46
	Random-balanced (1:1)	73.78	87.93±4.51	85.39±0.57
	All-tissue	100.0	89.24±1.61	86.57±0.69
	FTU-Seek	67.2	88.48±2.89	86.33±0.27

Random-balanced training used an equal number of randomly selected negative patches and positive patches. FTU-Seek Top1000 was used as a representative operating point for cross-task comparison. The same candidate Top*K* values were evaluated across the FTU categories to characterize the accuracy–workload trade-off under matched sampling budgets; these values were exploratory operating points and were not selected using a prevalence-adaptive rule or intended to define a universally optimal budget. Neither the BV nor the gland cohort included a held-out cohort; therefore, only development-set validation and internal-test results are reported. Workload denotes the proportion of retained training patches relative to all-tissue training.

**Table 7:** Segmentation performance across hard-negative budgets for blood-vessel and gland segmentation. FTU-Seek values are reported as mean ± SD across five folds, whereas Random values are summarized as mean ± SD across five random seeds, with each seed evaluated using five-fold models.

Task	*K*	FTU-Seek Validation	Random Validation (5 Seeds)	FTU-Seek Internal	Random Internal (5 Seeds)
BV	100	51.50±6.92	53.25±4.66	41.14±10.32	37.16±3.85
	300	57.03±5.35	58.63±3.29	47.93±11.80	47.23±6.78
	500	58.41±6.76	58.45±2.97	50.59±8.41	47.63±11.62
	1000	60.11±7.43	60.95±7.25	57.68±6.88	54.21±6.17
Gland	100	84.81±9.37	87.32±4.12	83.01±2.44	84.43±0.53
	300	87.78±3.38	87.06±3.90	85.13±0.38	84.70±0.98
	500	87.46±5.27	87.77±4.19	85.90±0.91	85.47±1.03
	1000	88.48±2.89	88.59±2.36	86.33±0.27	86.49±0.82

Random denotes random selection of the same number of target-absent patches per WSI as the corresponding FTU-Seek Top*K* configuration. Neither the BV nor the gland cohort included a held-out cohort; therefore, only development-set validation and internal-test results are reported.

**Table 8:** Comparison of representative TLS training-set construction strategies.

Strategy	Workload (%)	Development-Set Validation	Internal Test	Held-Out TLS Cohort
Positive-only	4.4	57.20±25.23	73.55±4.61	61.90±20.41
Random-balanced (1:1)	8.8	71.50±12.55	82.25±2.11	70.83±16.23
All-tissue	100.0	71.63±17.79	84.85±1.60	75.12±12.67
FTU-Seek	27.6	76.91±10.14	85.12±2.08	76.69±11.89

Random-balanced training used all positive patches and an equal number of negative patches randomly sampled from the target-absent patch pool, thereby maintaining a 1:1 positive-to-negative patch ratio. For the representative-strategy comparison, FTU-Seek used Top1000 as a conservative operating point for subsequent downstream analyses. The different *K* values were explored to characterize the accuracy–workload trade-off, rather than to define a universally optimal budget. The internal test and held-out TLS cohort were not used for configuration selection. Workload denotes the proportion of retained training patches relative to all-tissue training.

For TLS segmentation, positive-only training used 4.4% of the all-tissue workload but achieved only 73.55±4.61% independent-test Dice. Random-balanced training improved the test Dice to 82.25±2.11% with 8.8% workload, whereas all-tissue training reached 84.85±1.60%. FTU-Seek Top*K* sampling matched or exceeded the all-tissue test performance at three of the four evaluated budgets while using substantially fewer training patches. FTU-Seek Top100 achieved 85.08±1.07% Dice using 6.3% of the patches, and FTU-Seek Top500 achieved the highest independent-test Dice of 85.14±1.61% using 15.8% of the workload. Compared with matched random Top*K* sampling, FTU-Seek improved the internal-test Dice by 5.22, 3.81, 3.48, and 1.20 percentage points at Top100, Top300, Top500, and Top1000, respectively. The corresponding paired slide-level comparisons on the independent held-out TLS cohort are reported separately in Table 9.

**Table 9:** Paired slide-level comparisons between FTU-Seek and alternative training-set construction strategies on the held-out TLS cohort.

Comparison	FTU-Seek Dice	Comparator Dice	Mean Δ	95% CI	Paired *t*	Wilcoxon
FTU-Seek Top100 vs. Random Top100	73.99±13.87	71.41±15.43	+2.58	[1.14,4.02]	0.00096	0.00021
FTU-Seek Top300 vs. Random Top300	76.54±13.03	67.59±18.74	+8.96	[5.61,12.31]	<0.0001	<0.0001
FTU-Seek Top500 vs. Random Top500	75.19±13.54	70.48±16.84	+4.72	[3.06,6.38]	<0.0001	<0.0001
FTU-Seek Top1000 vs. Random Top1000	76.69±11.89	72.97±15.32	+3.72	[2.05,5.38]	<0.0001	<0.0001
FTU-Seek Top1000 vs. All-tissue	76.69±11.89	75.12±12.67	+1.57	[0.86,2.28]	<0.0001	<0.0001
FTU-Seek Top1000 vs. Random-balanced (1:1)	76.69±11.89	70.83±16.23	+5.86	[3.64,8.07]	<0.0001	<0.0001

For the held-out TLS cohort, Table 5 reports aggregate performance summaries across cross-validation folds and random seeds, whereas the present table reports WSI-level paired analyses. Specifically, Dice was calculated separately for each of the 30 held-out WSIs, with each WSI serving as the statistical pairing unit. Therefore, the present analysis estimates within-slide performance differences rather than reproducing the aggregate performance summaries reported in Table 5, and the corresponding mean values are not expected to be numerically identical.

BV segmentation showed a stronger dependence on negative-patch coverage. Positive-only training achieved only 32.14±3.98% test Dice, whereas all-tissue training achieved the best performance (60.38±9.76%). FTU-Seek performance increased as the hard-negative budget expanded, rising from 41.14±10.32% at Top100 to 57.68±6.88% at Top1000. The latter used 50.0% of the all-tissue workload and approached all-tissue performance while halving the retained training patches. FTU-Seek also outperformed matched random Top*K* sampling at all BV budgets, with test Dice gains of 3.98, 0.70, 2.96, and 3.47 percentage points from Top100 to Top1000.

For gland segmentation, all strategies achieved relatively high Dice scores, consistent with the greater abundance and more distinct morphology of glandular structures. All-tissue training remained the strongest baseline (86.57±0.69% test Dice), but reduced-workload strategies retained most of this performance. FTU-Seek Top300, Top500, and Top1000 achieved 85.13±0.38%, 85.90±0.91%, and 86.33±0.27%, respectively. FP-find Top1000 was within 0.24 percentage points of all-tissue training while reducing the workload to 67.2%. However, FP-find did not consistently outperform matched random Top*K* sampling across gland budgets, with random sampling performing comparably or better at several settings. These results indicate that FP-find offers no clear overall advantage in gland segmentation, likely because gland-containing patches are relatively abundant and morphologically easier to distinguish.

As summarized in Table 5, Table 6 and Table 7, the value of hard-negative mining was task-dependent. TLS benefited most from classifier-guided selection at small workloads, BV required broader negative coverage to approach all-tissue performance, and gland segmentation was comparatively insensitive to the negative-sampling strategy. Overall, FTU-Seek provided the clearest advantage for sparse or morphologically ambiguous FTUs, where randomly selected background patches are less likely to capture difficult false-positive regions.

### 3.4. Multi-Seed Stability of Random-Sampling Baselines

We next examined whether the performance of the random-sampling baselines depended strongly on a single random draw. Across TLS, blood-vessel, and gland segmentation, each random-sampling configuration was repeated using four additional seeds, resulting in five seeds in total when the original seed was included. The repeated runs showed limited-to-moderate variability across random seeds, with the magnitude depending on the FTU category and negative-patch budget. For TLS segmentation, the random Top100, Top300, Top500, and Top1000 baselines achieved mean Dice values of 79.86±3.45%, 80.88±1.55%, 81.66±1.24%, and 83.92±0.54%, respectively, on the internal two-WSI test cohort. The corresponding FP-find results from the original seed-1 experiments were 85.08%, 84.69%, 85.14%, and 85.12%. Although these comparisons should be interpreted cautiously because the internal test cohort contained only two WSIs, the FP-find results remained above the random-baseline averages at all four budgets.

The benefit was less pronounced for the denser FTU tasks. For blood-vessel segmentation, the differences between FP-find and the random-baseline averages were +3.98, +0.70, +2.96, and +3.47 percentage points from Top100 to Top1000, respectively. For gland segmentation, the corresponding differences were −1.42, +0.43, +0.43, and −0.16 percentage points. These results are consistent with the task-dependent behavior observed in the main experiments: random negative sampling may be sufficient when target-containing patches are relatively abundant and morphologically distinct, whereas classifier-guided selection is more advantageous for sparse or morphologically ambiguous targets.

### 3.5. Evaluation on an Independent Held-Out TLS Cohort

To determine whether the observed performance differences were preserved beyond the original two-slide internal test set, we evaluated all 11 segmentation strategies on an independent held-out cohort of 30 TLS WSIs from the same institution. The cohort included 30 patients and 30 WSIs, with 1184 annotated TLS instances, 123,484 tissue patches, and a TLS pixel-area ratio of 1.68% (Table 10). This cohort was not used during training, model selection, Top*K* selection, or threshold tuning. The evaluated strategies included positive-only training, all-tissue training, random-negative sampling, random Top*K* sampling, and classifier-guided FP-find Top*K* sampling.

On the held-out TLS cohort, FTU-Seek achieved slide-level Dice values of 73.99%, 76.54%, 75.19%, and 76.69% at Top100, Top300, Top500, and Top1000, respectively. The corresponding matched random Top*K* strategies achieved 71.41%, 67.59%, 70.48%, and 72.97%, respectively. The largest paired improvement was observed at Top300, whereas Top1000 achieved the highest absolute FTU-Seek Dice.

Based on the development-set analysis, Top1000 was retained as a pre-specified representative operating point for comparisons with all-tissue and random-balanced training. The held-out cohort was not used for selecting the operating point or tuning the segmentation procedure. Formal paired comparisons across the 30 held-out WSIs are reported in Table 9.

### 3.6. Paired Slide-Level Analysis on the Held-Out TLS Cohort

To assess whether the observed benefit of FTU-Seek was preserved in an independent held-out cohort, we performed paired slide-level analyses on the held-out TLS cohort. The same 30 WSIs were evaluated using FTU-Seek and the corresponding comparator strategy. For each WSI, the paired Dice difference was calculated as

$$\Delta_{i}={\mathrm{Dice}}_{\mathrm{FTU}\text{-}\mathrm{Seek},i}-{\mathrm{Dice}}_{\mathrm{Comparator},i},$$

where a positive value indicates better performance of FTU-Seek. Each model prediction was generated using a five-fold probability ensemble, in which the foreground probabilities from the five fold models were averaged before thresholding. The mean paired difference, its two-sided 95% confidence interval, paired *t*-test, and Wilcoxon signed-rank test were calculated across the 30 WSIs. The internal test cohort contained only two WSIs and was therefore excluded from formal slide-level hypothesis testing and retained for descriptive comparison only. The paired comparisons included matched random Top*K* sampling at all four budgets, as well as the representative Top1000 comparisons with all-tissue and random-balanced training.

Across all evaluated hard-negative budgets, FTU-Seek achieved higher slide-level Dice than matched random Top*K* sampling on the held-out TLS cohort. The mean paired improvements ranged from 2.58 to 8.96 percentage points, and all corresponding 95% confidence intervals excluded zero. At the representative Top1000 operating point, FTU-Seek also showed positive paired differences relative to all-tissue training (Δ=+1.57 percentage points) and random-balanced training (Δ=+5.86 percentage points). These results provide independent support for the generalization of FTU-Seek beyond the development data. The largest improvement over matched random sampling was observed at Top300, whereas Top1000 achieved the highest absolute FTU-Seek Dice.

### 3.7. Qualitative Analysis

**Figure 9:**
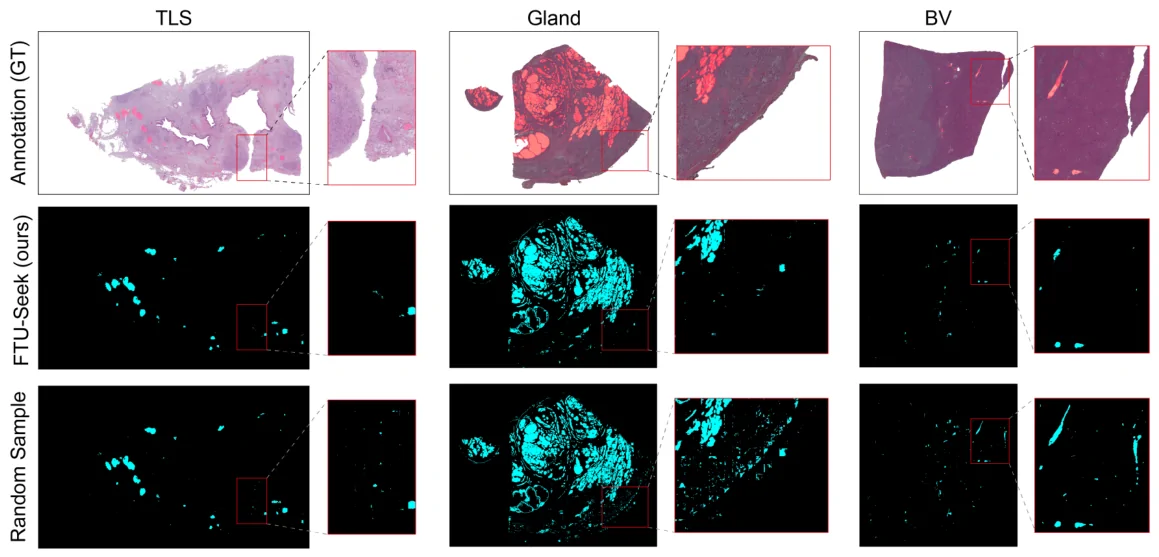
Visual comparison of segmentation performance across the three FTU cohorts. Representative results are shown for TLS, gland, and blood vessel segmentation using classifier-guided FTU-Seek and matched random negative sampling. Red boxes indicate the magnified regions used to highlight false-positive predictions and boundary discrepancies. GT, ground truth.

Figure 9 presents representative whole-slide segmentation results for the TLS, gland, and BV cohorts, together with magnified regions for detailed comparison. Across the three tasks, FTU-Seek produced segmentation patterns that were generally more consistent with the ground-truth annotations than those obtained using random negative sampling. The differences were most evident in regions containing sparse targets, morphologically ambiguous background structures, or complex boundaries.

For TLS segmentation, both methods identified the major TLS regions distributed across the tissue section. However, random negative sampling generated additional scattered predictions in background regions, particularly within the enlarged region, whereas FTU-Seek more effectively suppressed these isolated false-positive detections. At the same time, the principal TLS foci were retained, indicating that classifier-guided hard-negative selection improved background discrimination without substantially reducing sensitivity to sparse target regions.

For gland segmentation, the overall spatial distribution of glandular tissue was captured by both approaches. Nevertheless, random negative sampling produced a visibly denser and more fragmented prediction pattern in the magnified region, including responses extending into non-glandular tissue. In comparison, FTU-Seek yielded a cleaner segmentation map with fewer spurious structures and a distribution more closely aligned with the annotated gland regions. These observations are consistent with its ability to prioritize morphologically confusing negative patches during training.

The distinction between the two strategies was also apparent in BV segmentation. Although both methods detected major vascular structures, random negative sampling produced several elongated or punctate false-positive regions in the background. FTU-Seek suppressed many of these vessel-like confounders while preserving the principal annotated vessels. This behavior is particularly important for BV segmentation, where tissue clefts, elongated stromal structures, and other linear components may resemble vascular profiles.

Overall, the qualitative results support the quantitative findings in Table 5, Table 6 and Table 7 and Figure 9. Compared with random negative sampling, FTU-Seek primarily improved segmentation by reducing false-positive predictions and refining target boundaries, rather than by simply increasing the total predicted area. The benefit was especially evident for sparse or morphologically ambiguous FTUs, demonstrating that classifier-guided hard-negative sampling provides more informative background examples for segmentation model training.

### 3.8. Proof-of-Concept Downstream Phenotypic Analyses Enabled by FTU-Seek Segmentation

To illustrate the utility of FTU-Seek beyond segmentation performance, we converted segmentation masks from external TCGA cohorts into quantitative FTU phenotypes and examined their relationships with selected biological and clinicopathological characteristics. These proof-of-concept analyses were exploratory and were intended to demonstrate that FTU-Seek outputs can support downstream phenotyping and hypothesis generation, rather than to develop or validate clinical prediction models.

**Figure 10:**
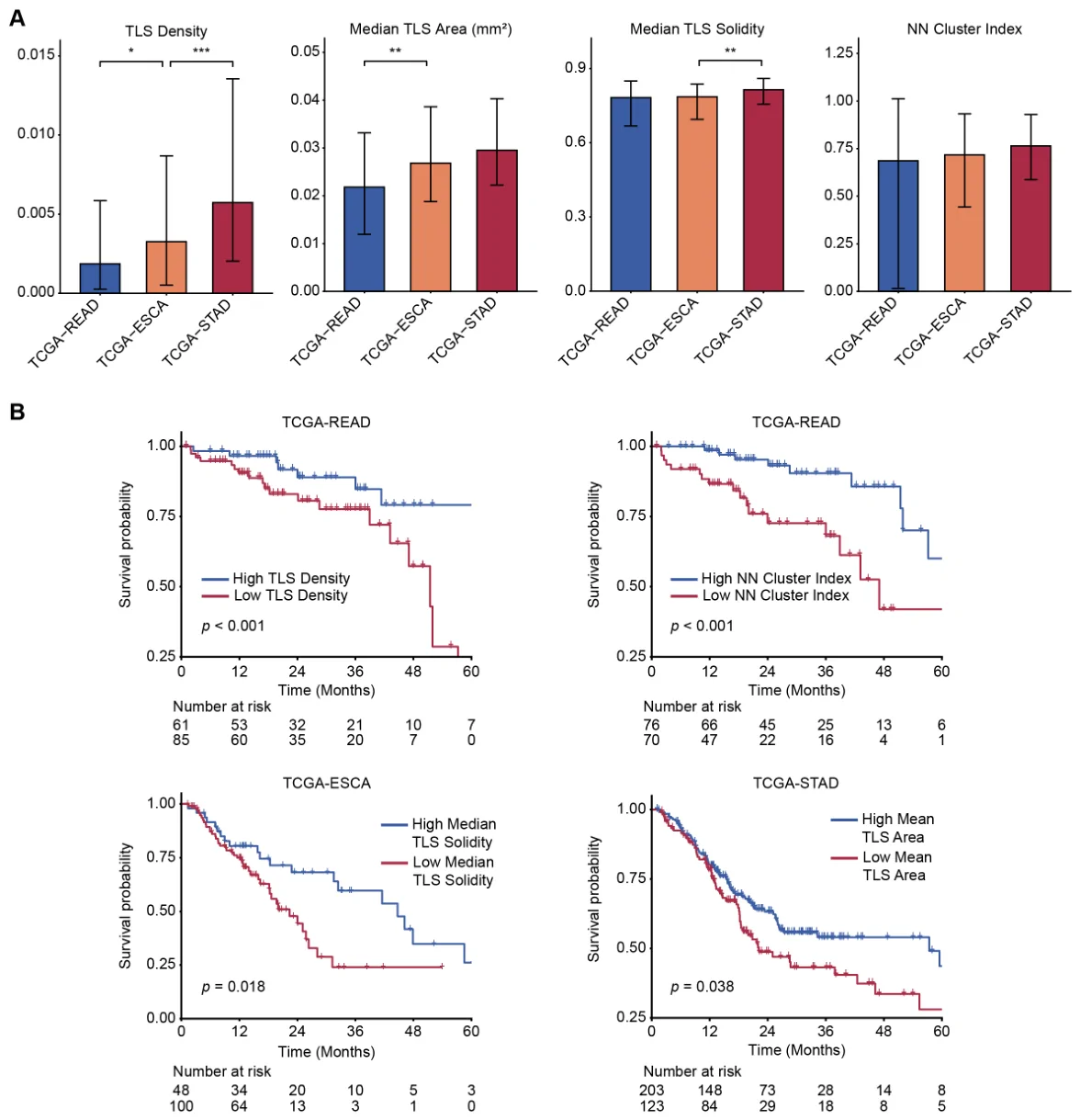
Exploratory characterization of TLS phenotypes and survival associations in TCGA cohorts. (**A**) Comparison of multi-dimensional TLS features across TCGA-READ, TCGA-ESCA, and TCGA-STAD. Panels from left to right present TLS Density, Median TLS area, median TLS solidity, and NN (nearest neighbor) cluster index. * *p* < 0.05, ** *p* < 0.01, and *** *p* < 0.001. (**B**) Kaplan–Meier survival analysis of key TLS features. Patients were stratified into high- and low-value groups based on exploratory data-derived cutoff values. Represented are TLS density and NN cluster index in the TCGA-READ cohort, median TLS solidity in the TCGA-ESCA cohort, and Mean TLS area in the TCGA-STAD cohort.

For TLSs, segmentation-derived phenotypes were quantified in TCGA-READ, TCGA-ESCA, and TCGA-STAD. Matched survival analyses included 146 READ, 148 ESCA, and 326 STAD patients after merging available WSI-derived TLS features with clinical outcomes. A total of 60 TLS features captured complementary aspects of immune organization, including TLS burden, size composition, hotspot density, and nearest-neighbor clustering. Across cohorts, mean TLS density ranged from 0.0048 in READ to 0.0116 in STAD, indicating substantial inter-cohort heterogeneity in TLS abundance (Figure 10A). Furthermore, TLS features exhibited significant heterogeneity across the different cohorts (Figure 10A). Specifically, the median instance-level TLS area in the READ cohort was significantly smaller than that in the other cohorts (*p* = 0.010). Conversely, TLSs in the STAD cohort demonstrated higher solidity (*p* = 0.001). In morphological analysis, solidity is defined as the ratio of the actual TLS area to the area of its convex hull, essentially reflecting a more compact structure with smoother, less irregular boundaries. Regarding spatial distribution, there were no significant differences across the three cohorts in the nearest neighbor (NN) cluster index. This spatial metric quantifies the degree of TLS clustering by comparing the observed average distance between adjacent TLSs against the expected distance under a completely random spatial distribution. Specifically, in READ, a 1-SD increase in TLS density was associated with a 51% reduction in the risk of death (HR = 0.49, 95% CI: 0.26–0.92; nominal p=0.026; FDR-adjusted p=0.25), whereas higher NN cluster index values were associated with a 60% lower risk (HR = 0.40, 95% CI: 0.17–0.95; nominal p=0.039; FDR-adjusted p=0.25). Neither association met the exploratory FDR threshold. For illustrative exploratory visualization, Kaplan–Meier curves were additionally generated for selected TLS phenotypes using data-derived cut points. These included TLS density and NN cluster index in READ, median TLS solidity in ESCA, and mean TLS area in STAD (Figure 10B). The resulting group separations were used solely to illustrate potential survival stratification and were not interpreted as validated or multiplicity-adjusted prognostic associations.

**Figure 11:**
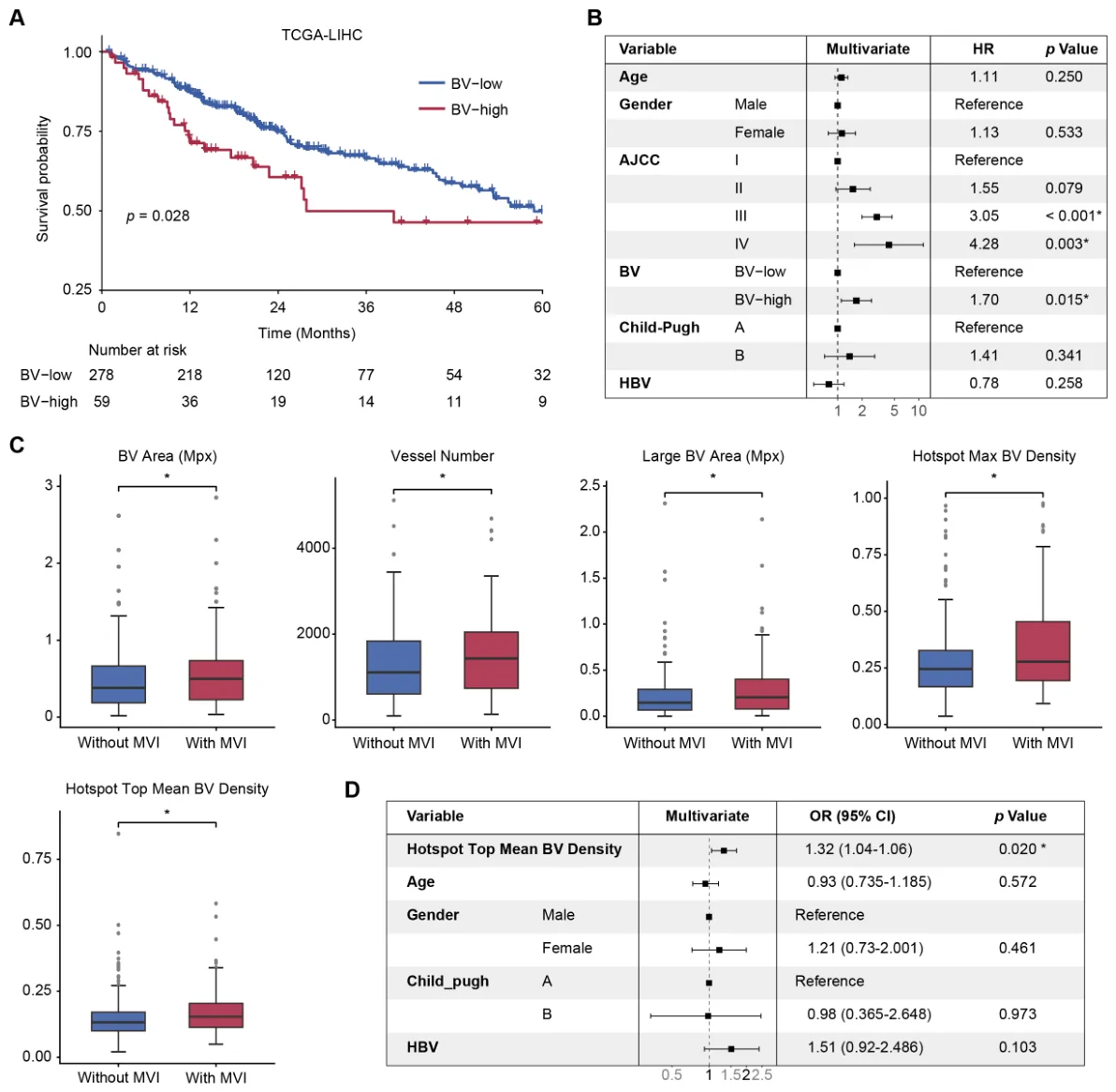
Exploratory associations of blood-vessel phenotypes with overall survival (OS) and microvascular invasion (MVI) in TCGA-LIHC. (**A**) Kaplan–Meier estimates of overall survival (OS) for patients stratified into BV-low (blue line) and BV-high (red line) groups based on an exploratory data-derived cutoff value. Statistical significance was assessed using the log-rank test. (**B**) Forest plot of the multivariable Cox proportional hazards regression analysis for OS. The model incorporates clinical covariates including age, gender, AJCC stage, BV classification (BV-low vs. BV-high), Child–Pugh score, and Hepatitis B Virus (HBV) status. Hazard ratios (HR) and corresponding *p*-values are presented. (**C**) Boxplots displaying the distribution of quantitative computational BV features in patient groups without and with MVI. The central horizontal line represents the median, box edges denote the interquartile range (IQR). (**D**) Forest plot presenting the multivariable logistic regression analysis for MVI status. The model evaluates hotspot top mean BV density and adjusts for baseline clinical characteristics (age, gender, Child–Pugh score, and HBV status). Odds ratios (ORs), 95% confidence intervals (CIs), and *p*-values are provided for each variable. * p<0.05.

For blood vessels, FTU-Seek-derived vascular phenotypes were quantified in TCGA-LIHC. The LIHC cohort included 337 patients and 121 death events. Among the 36 vascular phenotypes evaluated, higher BV count density showed a potential adverse association with OS (log-rank *p* = 0.028; Figure 11A). In the multivariable Cox analysis, the high-BV-density group showed a higher mortality risk than the low-density group after adjustment for age, clinical stage, Child–Pugh class, and HBV status (adjusted HR = 1.70, 95% CI: 1.10–2.59; *p* = 0.016; Figure 11B). The proportional-hazards diagnostic indicated evidence of a time-varying association, and the HR was therefore interpreted as an exploratory overall summary. No substantial multicollinearity was observed among the included predictors (all VIFs < 1.15). Furthermore, we observed significant differences in the distribution of computationally extracted vascular features—including BV area (nominal p=0.032; FDR-adjusted p=0.087), large BV area (nominal p=0.025; FDR-adjusted p=0.087), hotspot top mean BV density (nominal p=0.017; FDR-adjusted p=0.087), and hotspot max BV density (nominal p=0.011; FDR-adjusted p=0.087)—between patients with and without MVI (Figure 11C). Notably, in the multivariable logistic regression analysis, each 1-SD increase in hotspot top mean BV density—the mean BV density within the top decile of vascular hotspots—was associated with higher odds of MVI (adjusted OR = 1.32, 95% CI: 1.04–1.66; *p* = 0.020; Figure 11D). RCS analysis further supported an overall association between hotspot top mean BV density and MVI ($p_{\mathrm{overall}}$ = 0.001), with evidence of nonlinearity ($p_{\mathrm{nonlinearity}}$ = 0.015) and an overall trend toward higher odds of MVI at greater BV density. Overall, these analyses illustrate how FTU-Seek-derived vascular phenotypes can be used to explore potential relationships between tumor-associated vascular organization, mortality risk, and MVI.

**Figure 12:**
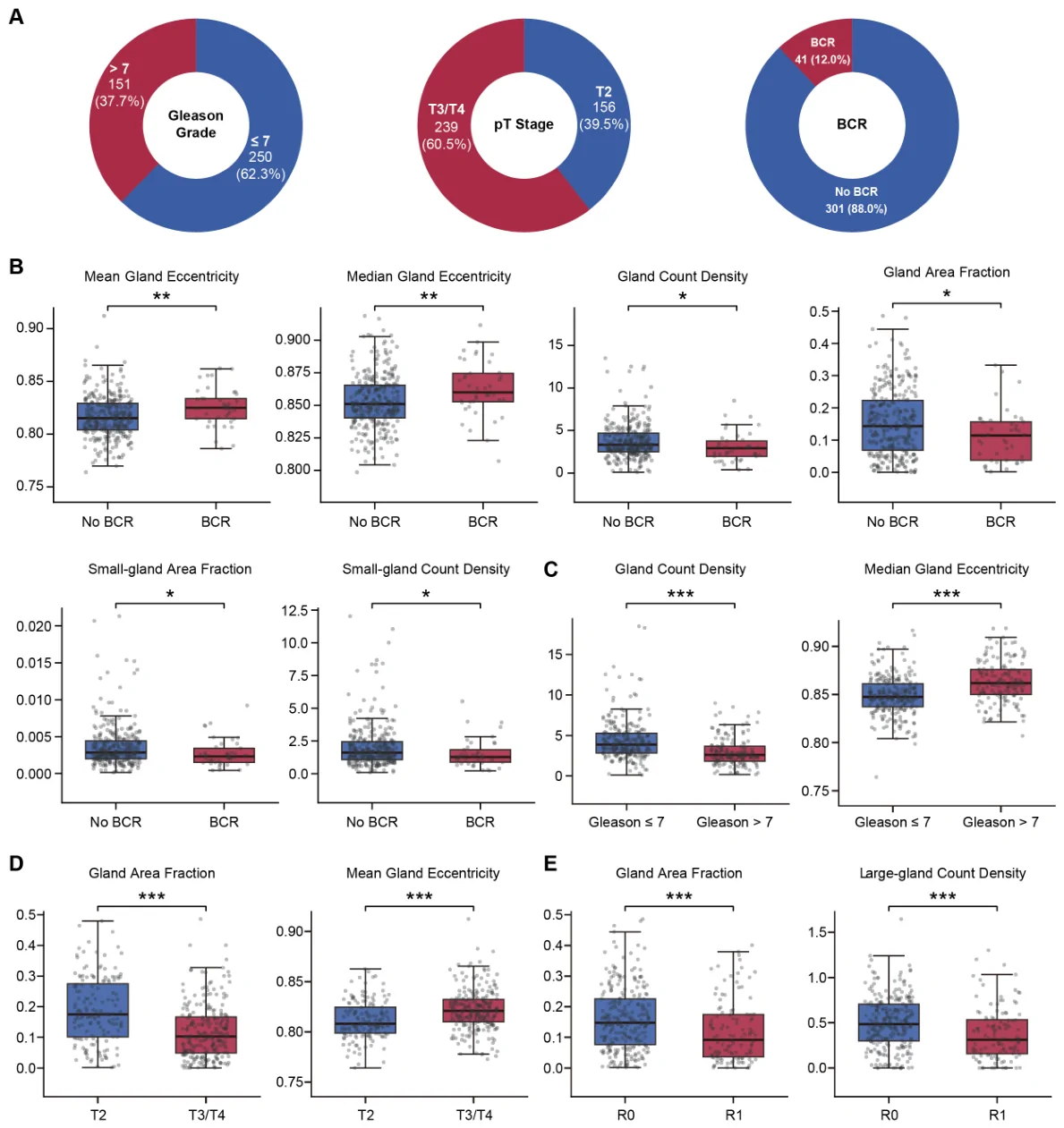
Association of segmented gland features with key clinicopathological determinants in the TCGA-PRAD cohort. (**A**) Donut charts illustrating the proportion of patients within the TCGA-PRAD cohort stratified by key clinical and pathological categories: Gleason grade (≤7 vs. >7), pT stage (T2 vs. T3/T4), and biochemical recurrence (BCR) status (No BCR vs. BCR). Slices indicate the count and percentage for each respective subgroup. (**B**–**E**) Boxplots detailing the distribution of extracted gland features that exhibited statistically significant differences between the respective clinical groups. (**B**) Distribution of gland features (mean gland eccentricity, median gland eccentricity, gland count density, gland area fraction, small-gland area fraction, and small-gland count density) compared between patients without and with BCR. (**C**) Comparison of gland count density and median gland eccentricity between Gleason grade ≤ 7 and >7 groups. (**D**) Distribution of gland area fraction and mean gland eccentricity across pT stage categories (T2 vs. T3/T4). (**E**) Comparison of gland area fraction and large-gland count density categorized by resection margin status (R0 vs. R1). For all boxplots, the central horizontal line represents the median, the box edges span the interquartile range (IQR). * nominal p<0.05, ** nominal p<0.01, *** nominal p<0.001.

FTU-Seek-derived 40 glandular architectural phenotypes were evaluated in TCGA-PRAD. The baseline clinical and pathological characteristics of the study cohort are summarized in Figure 12A. In terms of histological grading, the majority of patients presented with a Gleason score of ≤7 (n = 250, 62.3%), while 151 patients (37.7%) had a high-grade disease with a Gleason score of >7. Regarding pathological staging, a higher proportion of patients were diagnosed with advanced pT stage (T3/T4, n = 239, 60.5%) compared to localized stage (T2, n = 156, 39.5%). Furthermore, at the end of the follow-up period, BCR was observed in 41 patients (12.0%), with the remaining 301 patients (88.0%) staying BCR-free (Figure 12A). FTU-Seek-derived glandular architectural phenotypes were evaluated in TCGA-PRAD. In the BCR analysis, recurrent cases showed higher eccentricity and lower gland count density (nominal p<0.05; FDR-adjusted p<0.1; Figure 12B). Consistent with these findings, compared with tumors with a Gleason score ≤ 7, tumors with a Gleason score > 7 had lower gland count density (median 2.60 vs. 3.86 glands/mm^2^; FDR-adjusted p<0.001) and higher median gland eccentricity (median 0.862 vs. 0.847; FDR-adjusted p<0.001; Figure 12C). Similar trends were observed for local extension: pT3/T4 tumors showed lower gland area (median 0.103 vs. 0.175; FDR-adjusted p<0.001) and higher gland eccentricity (median 0.821 vs. 0.808; FDR-adjusted p<0.001) than pT2 tumors (Figure 12D). Additionally, positive surgical margins were associated with lower large-gland count density and lower total gland density (both FDR-adjusted p<0.001; Figure 12E). Collectively, these findings demonstrate that FTU-Seek-derived glandular phenotypes capture architectural alterations associated with adverse pathological characteristics and disease recurrence, supporting their utility for quantitative downstream tissue analysis.

## 4. Discussion

In this study, we developed FTU-Seek, a pathology foundation model (PFM)-guided hard-negative learning framework for sparse functional tissue unit (FTU) segmentation in whole-slide images (WSIs). Unlike conventional approaches that primarily employ foundation models as feature extractors or segmentation backbones, FTU-Seek additionally exploits pretrained histomorphological representations to construct morphology-aware segmentation training sets [47,48,68]. A patch-level classifier first ranks FTU-absent tissue according to its predicted target-containing probability, after which a static Top*K* strategy selects the most target-like negative patches for segmentation training. This design addresses a key challenge in quantitative histopathology: biologically meaningful microscopic structures are often sparsely distributed throughout large tissue sections, whereas the majority of tissue patches contain redundant background and only a limited subset comprises morphologically confusing regions likely to generate false-positive predictions. Across TLS, gland, and blood-vessel segmentation, our results show that morphology-aware negative selection improves the trade-off between segmentation accuracy and retained training workload, with the clearest benefits observed for sparse or morphologically ambiguous FTUs. The resulting segmentation outputs can further be transformed into quantitative tissue phenotypes for exploratory downstream analysis.

The segmentation experiments highlight that the composition of the negative training set is more important than its absolute size. Training using only FTU-containing patches consistently resulted in inferior performance, confirming that representative negative tissue is indispensable for learning robust decision boundaries. Conversely, all-tissue training provides abundant negative information but requires processing a very large number of easy background patches that contribute little additional supervision. FTU-Seek addresses this imbalance by preferentially selecting classifier-ranked target-like negatives, thereby concentrating training on the background regions most likely to generate false-positive predictions. Although this concept shares the objective of hard-example mining, FTU-Seek differs fundamentally from conventional online hard-example mining because difficult negatives are identified before segmentation training using frozen pathology foundation model representations rather than repeatedly re-mined during optimization. Consequently, the proposed strategy is computationally reproducible, avoids repeated screening of the entire WSI patch pool, and enables direct comparison with alternative sampling strategies under identical negative-sampling budgets. The reduction in training workload should be distinguished from a reduction in peak GPU-memory usage. FTU-Seek decreases the total number of retained training patches and therefore improves training-data efficiency without requiring additional GPU memory or a larger computational platform. However, when the model architecture, batch size, input resolution, and hardware configuration are fixed, reducing the total number of training patches does not necessarily reduce the peak GPU-memory usage per iteration. Accordingly, the computational benefit of FTU-Seek should be interpreted as improved training-data efficiency under a fixed memory budget rather than as a proportional reduction in GPU-memory consumption. A complete assessment of computational efficiency would additionally require systematic measurements of wall-clock training time, GPU utilization, peak memory, and energy consumption.

The task-dependent benefit of classifier-guided hard-negative selection may be explained by differences in target sparsity, class imbalance, and morphological ambiguity. When targets are sparse and imbalance is severe, the negative pool is dominated by easy background, making random sampling less likely to capture the small subset of target-like negatives most responsible for false-positive predictions. TLSs represented the sparsest segmentation task and exhibited the greatest efficiency gains. A relatively small number of classifier-selected negatives was sufficient to recover or slightly exceed the performance of all-tissue training, suggesting that only a limited subset of inflammatory or stromal regions contributes substantially to false-positive TLS predictions. Blood-vessel segmentation demonstrated a different behaviour. Because vessel-like structures occur in diverse morphological contexts, including tissue clefts, empty lumina, collagen bundles, elongated stromal regions, and red blood cell-rich spaces, segmentation performance continued to improve as larger hard-negative pools were incorporated. Gland segmentation was comparatively insensitive to the sampling strategy because gland-containing patches were considerably more abundant, resulting in less severe class imbalance, and the distinction between positive and negative tissue was substantially clearer. Collectively, these findings indicate that sparse FTUs derive greater benefit from targeted hard-negative selection, whereas more abundant FTUs with clearer class separation show smaller gains; broader morphological ambiguity may additionally require greater negative coverage. This task-dependent sampling strategy also provides a practical means of reducing redundant training data. By removing easy-negative patches before segmentation training, FTU-Seek reduces the number of samples processed during optimization while keeping the segmentation architecture, batch size, input resolution, and hardware configuration unchanged.

The classifier probability distributions provide further insight into the mechanism underlying FTU-Seek. For TLSs, the vast majority of negative patches received probabilities close to zero, while only a small high-probability tail corresponded to localized target-like tissue. Such distributions naturally favour strict Top*K* selection because informative negatives are concentrated within a relatively small subset. Blood vessels exhibited substantially broader overlap between positive and negative probability distributions, reflecting widespread morphological ambiguity and explaining why larger negative budgets were beneficial. Gland classification showed the clearest overall separation, although intermediate-probability regions remained around gland boundaries, pseudo-glandular spaces, epithelial debris, and fibrotic cavities. These observations indicate that classifier probabilities capture biologically meaningful morphological ambiguity rather than merely reflecting classification confidence. Future developments may therefore combine probability-based ranking with diversity constraints, uncertainty estimation, or tissue-context clustering to obtain even more representative hard-negative sets.

The patch-level classifier is an important intermediate component, but its accuracy does not guarantee improved segmentation. Because all target-containing patches are retained for segmentation training, classifier errors on positive patches do not directly remove positive training examples. In contrast, errors within the target-absent pool alter the composition of the selected hard-negative set. If a morphologically confusing negative patch receives an underestimated target-containing probability, it may be omitted from Top*K* selection, leaving the corresponding false-positive pattern insufficiently represented during segmentation training. Conversely, overestimated probabilities may allocate Top*K* slots to less informative negatives. Although such selections may still increase the diversity of background examples, extensive misranking can reduce the efficiency of hard-negative sampling. Thus, the quality and calibration of the patch-level classifier affect segmentation indirectly through negative-pool composition. Uncertainty-aware ranking or iterative negative re-mining may help mitigate this error propagation, but would introduce additional computational cost and potential variability.

An important component of FTU-Seek is the use of frozen pathology foundation model representations. Recent pathology foundation models such as UNI and Virchow have demonstrated remarkable transferability across computational pathology tasks by learning general histomorphological representations from large and heterogeneous pathology image collections [47,48,69]. In the present framework, these representations serve two complementary purposes. First, they provide robust patch-level features for identifying target-like negative tissue. Second, they supply transferable multi-scale representations for dense segmentation across structurally distinct FTUs. Freezing the encoder substantially reduces the number of trainable parameters while minimizing dependence on relatively small manually annotated datasets. Our results therefore suggest that pathology foundation models can contribute not only through transferable feature extraction but also through intelligent construction of segmentation training data, thereby extending their role beyond conventional transfer learning.

Beyond computational efficiency, FTU-Seek enables quantitative characterization of tissue architecture. TLSs, blood vessels, and glands represent complementary components of the tissue microenvironment that reflect adaptive immune organization, angiogenesis and vascular remodeling, and epithelial differentiation, respectively. In this study, their segmentation masks were converted into quantitative spatial phenotypes for exploratory downstream analyses.

Our proof-of-concept downstream analyses further illustrate this potential. Quantitative TLS features spanning abundance, spatial organization, and morphology exhibited variation across tumor types. The observed differences among READ, ESCA, and STAD are biologically plausible because TLS maturation, localization, cellular composition, and interactions with the surrounding tumor microenvironment differ substantially across tumor types [70,71,72,73]. Several TLS phenotypes also showed potential associations with OS when evaluated as continuous measures, supporting their utility for generating hypotheses regarding the relationship between immune architecture and patient outcome. Similarly, vascular phenotyping in hepatocellular carcinoma identified candidate features related to vessel density and localized vascular hotspots, with exploratory associations observed for OS and MVI. Finally, in prostate cancer, glandular phenotypes captured architectural alterations associated with adverse clinicopathological characteristics, including reduced gland density and increased eccentricity in more aggressive tumors. Collectively, these observations suggest that FTU-Seek-derived phenotypes retain information beyond the segmentation masks themselves and can serve as quantitative descriptors for subsequent biological and clinical investigation.

Several limitations should be acknowledged. First, although each development cohort contained extensive pixel-level annotations, the number of annotated WSIs remained relatively limited, and the internal test cohort contained only two WSIs per segmentation task. Accordingly, the internal-test results should be interpreted as descriptive evidence rather than definitive estimates of generalization. To provide a stronger independent assessment, we additionally evaluated the TLS segmentation strategies on 30 held-out WSIs from the same institution. However, this cohort does not constitute multi-center external validation. Larger multi-institutional studies will be necessary to evaluate the robustness of FTU-Seek across diverse patient populations, staining protocols, scanners, and tissue-processing procedures [74].

The additional held-out analysis showed that FTU-Seek achieved higher slide-level Dice than matched random Top*K* sampling across all four evaluated negative-patch budgets, with positive paired differences and confidence intervals excluding zero. These results support the robustness of the method within the available same-institution data setting, while broader multi-institutional validation remains necessary.

Second, the present workload metric was defined as the proportion of retained training patches relative to all-tissue training rather than direct measurements of computational time, GPU utilization, memory consumption, or energy efficiency. Future studies should therefore include comprehensive computational benchmarking. Third, the proposed static Top*K* strategy primarily emphasizes false-positive suppression and does not explicitly incorporate false-negative regions, annotation uncertainty, or difficult target boundaries. Iterative re-mining, uncertainty-guided sampling, boundary-aware optimization, and combined false-positive/false-negative mining may further improve segmentation performance. Fourth, identical Top*K* candidate values were evaluated across all FTU categories, although our results indicate that the optimal hard-negative budget depends on target prevalence and morphological complexity.

The fixed per-WSI allocation also means that slides with different tissue areas do not contribute negative patches in proportion to their available tissue area. Proportional allocation and globally ranked negative-patch selection were not evaluated in the present study. These alternative allocation schemes, together with prevalence- or uncertainty-adaptive budgets, remain directions for future work.

Adaptive sampling strategies based on classifier uncertainty, probability distributions, or validation performance may therefore provide greater flexibility. Finally, FTU-Seek may have potential future applications in the analysis of stem cell-derived tissues, organoids, and regenerative medicine specimens; however, these settings were not investigated in the present study and would require dedicated validation using appropriately annotated datasets.

The downstream TCGA analyses should be interpreted as exploratory and hypothesis-generating. Because manually annotated reference masks were unavailable, the TCGA cohorts were used for exploratory downstream application rather than direct external validation of segmentation performance. These retrospective analyses were not designed for biomarker validation, and the data-derived survival cut points should not be considered validated prognostic thresholds. Accordingly, the findings primarily demonstrate the feasibility of deriving quantitative phenotypes from FTU-Seek segmentations. Future integration with spatial transcriptomics, proteomics, and molecular pathology may further clarify the biological basis of these phenotypes and identify reproducible architectural features across disease contexts.

Overall, FTU-Seek extends pathology foundation models beyond transferable feature representation by using pretrained histomorphological information to identify target-like negative tissue before segmentation training and construct compact, morphology-aware training sets. This pre-segmentation hard-negative selection reduces redundant background sampling and retained training workload while maintaining competitive segmentation performance, with the clearest practical benefit observed for sparse and imbalanced FTUs. These findings should be interpreted in light of the relatively small number of annotated WSIs, the lack of direct wall-clock and resource benchmarking, and the dependence of the static Top*K* strategy on classifier-based ranking and task-specific sampling budgets. Larger multi-institutional cohorts, systematic computational benchmarking, and adaptive or iterative negative-selection strategies will be needed to establish the robustness and practical clinical applicability of FTU-Seek.

## 5. Conclusions

Overall, FTU-Seek extends pathology foundation models beyond feature representation by using pretrained histomorphological information to identify target-like negative tissue before segmentation training and construct compact, morphology-aware training sets. This pre-segmentation hard-negative selection directly addresses the challenge of sparse FTU segmentation by reducing redundant background sampling and retained training workload while maintaining competitive segmentation performance, with particularly clear benefits for sparse and imbalanced FTUs. The present study is limited by the relatively small number of annotated WSIs and the absence of direct computational benchmarking, while the static TopK strategy remains dependent on classifier-based ranking and task-specific sampling requirements. Prospective validation in independent multi-institutional cohorts will therefore be required before the clinical applicability of FTU-Seek can be established, and more adaptive sampling strategies warrant further investigation.

## Figures and Tables

**Table 1:** Dataset characteristics and spatial sparsity of the three FTU segmentation cohorts at instance, patch, and pixel levels.

Characteristics	TLS	BV	Gland
Development Set			
Instance level			
Unique patients	8	7	8
WSIs	8	7	8
WSIs per patient	1	1	1
Total FTU instances	133	2508	3074
Mean FTU instances per WSI	16.62	358.29	384.25
Median FTU instances per WSI	12.50	316.00	377.50
Patch level			
Total tissue patches	33,752	20,831	26,366
FTU-containing patches	1322	3422	9719
FTU-absent patches	32,430	17,409	16,647
FTU-containing patch ratio (%)	3.92	16.43	36.86
Pixel level			
Tissue pixel area	145,723,284	67,053,366	80,370,006
FTU pixel area	942,758	1,646,695	15,197,324
FTU pixel area ratio (%)	0.65	2.46	18.91
Test Set			
Instance level			
Unique patients	2	2	2
WSIs	2	2	2
WSIs per patient	1	1	1
Total FTU instances	105	409	453
Mean FTU instances per WSI	52.50	204.50	226.50
Median FTU instances per WSI	52.50	204.50	226.50
Patch level			
Total tissue patches	9421	5222	5432
FTU-containing patches	757	583	1951
FTU-absent patches	8664	4639	3481
FTU-containing patch ratio (%)	8.04	11.16	35.92
Pixel level			
Tissue pixel area	38,848,017	16,370,435	16,686,067
FTU pixel area	901,489	273,117	3,981,224
FTU pixel area ratio (%)	2.32	1.67	23.86

Abbreviations: FTU, functional tissue unit; TLS, tertiary lymphoid structure; BV, blood vessel; WSI, whole-slide image. Dataset statistics were calculated from the actual slides included in the train_val_test.json split files used for segmentation experiments. Development and test set statistics are reported separately in this table and were not pooled. “FTU-containing patches” denotes tissue patches overlapping annotated FTU regions, whereas “FTU-absent patches” denotes tissue patches without annotated FTU regions. Patch-level ratios were calculated as FTU-containing patches divided by total tissue patches. Pixel-level ratios were calculated as FTU pixel area divided by total tissue pixel area.

**Table 2:** Baseline characteristics of the TCGA cohorts.

	TCGA-	TCGA-	TCGA-	TCGA-	TCGA-
	STAD	READ	ESCA	LIHC	PRAD
	(n=326)	(n=146)	(n=148)	(n=337)	(n=401)
Sex					
Male	215 (65.95)	82 (56.16)	128 (86.49)	228 (67.66)	401 (100)
Female	111 (34.05)	64 (43.84)	20 (13.51)	109 (32.34)	0 (0)
Age	66 (57–72)	65 (57–72)	59 (53–69)	61 (51–68)	61 (56–66)
Stage					
I	39 (11.96)	28 (19.18)	34 (22.97)	166 (49.26)	29 (7.23)
II	109 (33.44)	46 (31.51)	49 (33.11)	81 (24.04)	121 (30.17)
III	159 (48.77)	51 (35.62)	47 (31.76)	83 (24.63)	192 (47.88)
IV	19 (5.83)	21 (13.70)	18 (12.16)	7 (2.08)	59 (14.71)
Median Follow-up	26.68	25.03	20.01	28.88	34.83
(months, 95% CI)	(22.67–29.57)	(20.53–30.98)	(14.85–25.20)	(25.07–35.06)	(31.67–37.95)
Status					
Alive	194 (59.51)	120 (82.19)	86 (58.11)	216 (64.09)	391 (97.51)
Dead	132 (40.49)	26 (17.81)	62 (41.89)	121 (35.91)	10 (2.49)
Survival Rate (%)					
1-year	79.52	93.19	76.87	84.92	99.73
3-year	50.22	82.86	40.57	63.73	98.26
5-year	37.24	49.60	19.13	49.67	97.61

Data are n (%) or median (IQR). TCGA, The Cancer Genome Atlas. Stage is based on the 8th edition of AJCC staging.

**Table 3:** External TCGA cohorts used for exploratory downstream application.

FTU Task	TCGA Cohort	Analysis Endpoint	Matched Patients	Events/Groups
TLS	READ	Overall survival	146	26 deaths
TLS	ESCA	Overall survival	148	62 deaths
TLS	STAD	Overall survival	326	132 deaths
BV	LIHC	Overall survival	337	121 deaths
BV	LIHC	MVI	337	100 positive vs. 237 negative
Gland	PRAD	Gleason score	401	151 high vs. 250 low
Gland	PRAD	Pathological T stage	395	239 T3/T4 vs. 156 T2
Gland	PRAD	Surgical margin	372	115 R1 vs. 257 R0
Gland	PRAD	BCR status	342	301 non-recurrent

Abbreviations: BCR, biochemical recurrence; BV, blood vessel; ESCA, esophageal carcinoma; LIHC, liver hepatocellular carcinoma; FTU, functional tissue unit; PRAD, prostate adenocarcinoma; READ, rectum adenocarcinoma; STAD, stomach adenocarcinoma; TCGA, The Cancer Genome Atlas; TLS, tertiary lymphoid structure.

**Table 4:** Validation-best patch-level classifier performance across five-fold cross-validation. AUC is the primary metric. Values are percentages.

Cohort	Fold	AUC	Sensitivity	Specificity	FPR	NPV	PPV
TLS	0	92.02	88.04	81.94	18.06	99.72	8.52
	1	95.70	87.47	91.63	8.37	99.28	35.79
	2	97.97	90.77	97.05	2.95	99.37	67.14
	3	99.19	94.44	98.27	1.73	99.87	55.43
	4	94.70	81.56	91.16	8.84	99.16	27.98
	Mean ± SD	95.92 ± 2.81	88.46 ± 4.74	92.01 ± 6.46	7.99 ± 6.46	99.48 ± 0.30	38.97 ± 23.04
BV	0	87.50	75.00	85.62	14.38	93.93	53.60
	1	91.83	82.50	85.36	14.64	97.59	40.39
	2	94.28	88.83	85.53	14.47	96.80	60.83
	3	90.26	89.12	76.12	23.88	96.19	50.84
	4	86.70	81.99	75.35	24.65	95.55	39.32
	Mean ± SD	90.11 ± 3.11	83.49 ± 5.82	81.60 ± 5.36	18.40 ± 5.36	96.01 ± 1.39	49.00 ± 9.11
Gland	0	98.75	92.98	96.64	3.36	94.30	95.84
	1	98.25	92.64	96.01	3.99	95.29	93.74
	2	97.66	91.20	95.15	4.85	97.71	82.64
	3	98.73	94.03	95.83	4.17	97.24	91.13
	4	97.41	91.62	94.56	5.44	89.54	95.69
	Mean ± SD	98.16 ± 0.61	92.49 ± 1.12	95.64 ± 0.80	4.36 ± 0.80	94.82 ± 3.26	91.81 ± 5.47

**Table 10:** Characteristics and spatial sparsity of the independent held-out TLS cohort.

Characteristic	Value
Patients	30
WSIs	30
Total TLS instances	1184
Mean TLS instances per WSI	39.47
Median TLS instances per WSI	33.00
Total tissue patches	123,484
TLS-containing patches	9231
TLS-absent patches	114,253
TLS-containing patch ratio (%)	7.48
Tissue pixel area	525,755,637
TLS pixel area	8,822,896
TLS pixel area ratio (%)	1.68

The cohort contained one WSI per patient. Tissue patches were extracted at 1 μm/pixel with a nominal patch size of 256×256 pixels before resizing to the model input resolution. TLS-containing patches overlapped at least one annotated TLS region, whereas TLS-absent patches did not overlap an annotated TLS region. Pixel areas were calculated from the corresponding tissue and TLS annotation masks.

## Data Availability

All diagnostic whole-slide images in TCGA cohorts and corresponding clinical information are publicly accessible through the NIH Genomic Data Commons Data Portal (https://portal.gdc.cancer.gov). The annotations and clinicopathological data from FCH cohorts contain sensitive patient information and are not publicly available. The source code, split metadata, configuration files, and model weights that can be legally shared will be released upon publication. Data are made available exclusively for noncommercial academic research, subject to ethical consent. Access requests should be directed to the corresponding author (J.L.) and require a brief research proposal and execution of a data use agreement.

## Abbreviations

BCR, biochemical recurrence; BV, blood vessel; CI, confidence interval; DSC, dice similarity coefficient; ESCA, esophageal carcinoma; FN, false negative; FP, false positive; FTU, functional tissue unit; H&E, hematoxylin and eosin; HR, hazard ratio; IQR, interquartile range; LIHC, liver hepatocellular carcinoma; MVI, microvascular invasion; OR, odds ratio; OS, overall survival; PFM, pathology foundation model; PRAD, prostate adenocarcinoma; READ, rectum adenocarcinoma; SD, standard deviation; STAD, stomach adenocarcinoma; TCGA, The Cancer Genome Atlas; TLS, tertiary lymphoid structure; WSI, whole-slide image.
